# Palmitic Acid Accelerates Endothelial Cell Injury and Cardiovascular Dysfunction via Palmitoylation of PKM2

**DOI:** 10.1002/advs.202412895

**Published:** 2024-12-12

**Authors:** Yu He, Senlin Li, Lujing Jiang, Kejue Wu, Shanshan Chen, Linjie Su, Cui Liu, Peiqing Liu, Wenwei Luo, Shilong Zhong, Zhuoming Li

**Affiliations:** ^1^ Department of Pharmacology and Toxicology School of Pharmaceutical Sciences National and Local United Engineering Lab of Druggability and New Drugs Evaluation Guangdong Engineering Laboratory of Druggability and New Drug Evaluation Guangdong Provincial Key Laboratory of New Drug Design and Evaluation Sun Yat‐sen University Guangzhou 510006 P. R. China; ^2^ Department of Pharmacy Guangdong Provincial People's Hospital (Guangdong Academy of Medical Sciences) Southern Medical University Guangzhou 510080 P. R. China; ^3^ Guangdong Provincial Key Laboratory of Coronary Heart Disease Prevention Guangdong Cardiovascular Institute Guangdong Provincial People's Hospital Guangdong Academy of Medical Sciences Guangzhou 510080 P. R. China; ^4^ School of Medicine South China University of Technology Guangzhou 510006 P. R. China

**Keywords:** cardiovascular dysfunction, endothelial injury, palmitic acid, palmitoylation, PKM2

## Abstract

High serum level of palmitic acid(PA) is implicated in pathogenesis of cardiovascular diseases. PA serves as the substrate for protein palmitoylation. However, it is still unknown whether palmitoylation is involved in PA‐induced cardiovascular dysfunction. Here, in clinical cohort studies of 1040 patients with coronary heart disease, high level of PA is associated with risk of major adverse cardiovascular events (MACE) and death. In ApoE^−/−^mice, 10 mg/kg^−1^ PA treatment induces blood pressure elevation, cardiac contractile dysfunction, endothelial dysfunction and atherosclerotic plaqueformation. In endothelial cells, inhibition of palmitoylation bypalmitoyl‐transferase inhibitor 2‐BP eliminates PA‐induced endothelial injury, whereas promotion of palmitoylation by depalmitoylase inhibitor ML349 exacerbates the harmful effect of PA. Palmitoyl‐proteomics analysis identifies pyruvate kinase isozyme type M2 (PKM2) as the palmitoylated protein responsible for PA‐induced endothelial injury, and Cys31 as the predominant palmitoylated site. PKM2‐C31S mutants (cysteine replaced by serine) prevents PA‐induced endothelial injury. Endothelial‐specific AAV‐C31S PKM2^endo^ ameliorates cardiovascular dysfunction caused by PA in ApoE^−/−^ mice. Mechanistically, PKM2‐C31 palmitoylation impairs PKM2 tetramerization to inhibit its pyruvate kinase activity and endothelial glycolysis. Finally, zDHHC13 is identified as the palmitoyl acyltransferase of PKM2. In conclusion, these findings suggest that PKM2‐C31 palmitoylation contributes to PA‐induced endothelial injury and cardiovascular dysfunction.

## Graphical abstract

Clinical cohort studies indicate that high serum level of palmitic acid (PA) is associated with risk of cardiovascular events and death. The detrimental effect of PA is attributed to palmitoylation of pyruvate kinase isozyme type M2 (PKM2) at C31 in vascular endothelial cells. PKM2‐C31 palmitoylation impairs PKM2 tetramerization and inhibits endothelial glycolysis, finally resulting to endothelial cell injury and facilitating the development of cardiovascular diseases.

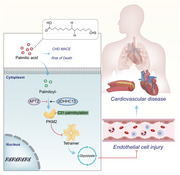

## 1 Introduction

Growing evidences indicate that lipid metabolites are pivotal regulators of biological function and cell signaling.^[^ ^1^, ^2^ ^]^ Abnormal lipid metabolism plays a key role in the pathogenesis of various diseases, in particular cardiovascular disease (CVD).^[^ ^3^, ^4^ ^]^ Palmitic acid (C16:0, PA) is the most abundant saturated fatty acid (FA) in daily diet and also comprises ≈20%–30% of total FA in membrane phospholipids and triglycerides in human.^[^ ^5^ ^]^ Plasma PA can be derived from dietary fat and also synthesized endogenously from *de novo* lipogenesis. Emerging epidemiological studies reveal that chronic high serum level of PA is associated with coronary heart disease (CHD), stroke, and metabolic syndrome, indicating that PA is a high risk factor for CVD.^[^ ^6^, ^7^, ^8^, ^9^ ^]^ However, mechanisms underlying the cardio‐detrimental effect of PA are still under explored.

In addition to serving as an energy source, PA may serve as the substrate for protein palmitoylation. PA is converted to palmitoyl‐CoA and then incorporates into cysteine residues of proteins through thioester bonds in a reversible process called S‐palmitoylation. S‐palmitoylation is catalyzed by a family of palmitoyl acyltransferases (PATs) named zDHHCs, which contain conserved zinc‐finger domain and Asp‐His‐His‐Cys (DHHC) motif.^[^ ^10^ ^]^ On the contrary, depalmitoylation that removes palmitate from palmitoylated proteins is catalyzed by a family of serine hydrolases, including acyl‐protein thioesterases (APT1, APT2), palmitoyl protein thioesterases (PPT1, PPT2), the α/β hydrolase domain proteins (ABHD).^[^ ^11^ ^]^ Protein palmitoylation acts as a switch to dynamically control the “turn on” and “turn off” of protein biological functions. By enhancing the hydrophobicity of proteins, palmitoylation participates in the regulation of protein structure and stability, enzymatic activity, subcellular localization and trafficking, as well as protein‐protein interaction. Aberrant protein palmitoylation is associated with the development of a variety of diseases. For instance, increased palmitoylation of fatty acid translocase CD36 promotes its localization on the plasma membrane of hepatocytes, thus enhancing FA uptake and lipid accumulation, finally contributing to non‐alcoholic steatohepatitis;^[^ ^12^ ^]^ palmitoylation of myeloid differentiation primary response protein (MYD88), due to *de novo* FA synthesis, activates the downstream inflammatory signaling in sepsis.^[^ ^13^ ^]^ Nevertheless, it is still unknown whether or not palmitoylation is involved in cardiovascular dysfunction in response to elevated PA level.

In the present study, we demonstrated that PA facilitated the palmitoylation of pyruvate kinase isozyme type M2 (PKM2), the key rate‐limiting enzyme responsible for catalyzing the final step of glycolysis, in vascular endothelial cells (ECs). Suppression of PKM2 palmitoylation elevated its enzymatic activity and improved endothelial glycolysis, ultimately conferred protection against PA‐induced endothelial cell injury and cardiovascular dysfunction. The novelty of this study lies in its demonstration of the mediating role of protein palmitoylation in PA‐induced CVD, which might suggest the potential role of PA as a clinical biomarker and suggest potential therapeutic strategies of CVD associated with abnormal lipid metabolism.

## 2 Results

### 2.1 Clinical Cohort Studies Revealed that High Level of PA was Associated with Adverse Cardiovascular Events and Risk of Death in CHD Patients

**Figure 1:**
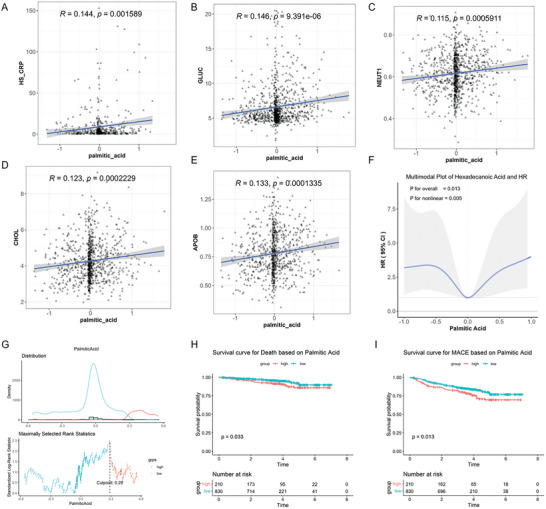
PA was positively associated with inflammation, glucolipid metabolism, and risk of death in coronary heart disease patients clinical cohort. 1040 patients with CHD were involved in this cohort. A‐E) Pearson correlation analysis and univariate linear regression analysis were employed to investigate the correlation between serum palmitic acid concentration and metabolites levels in patients with CHD (n = 1040). F) Restricted cubic splines were utilized to explore the nonlinear relationship between palmitic acid and the risk of mortality in CHD patients, and the nonlinear model was then analyzed using a Cox proportional hazards model (n = 1040) G) Patients were classified into low PA and high PA groups by a cutoff value (n = 1040). H,I) The log‐rank test was used to indicated the risk of death and MACE, as well as the Kaplan‐Meier survival curves demonstrated survival rates within low PA and high PA groups (n = 1040).

Aiming to clarify the relationship between palmitic acid levels and CVD, we conducted a baseline characteristic analysis of 1040 patients with CHD. The association between PA and blood biochemical indicators, biomarkers associated with CVD, risk of mortality, as well as major adverse cardiovascular events (MACE) which were defined as including all‐cause death, nonfatal myocardial infarctions, coronary revascularization, and cerebral infarction, were analyzed. As shown in Table S1 (Supporting Information), the prevalence of dyslipidemia in the cohorts was 72.54%. In laboratory examinations, the average fasting blood glucose (GLUC) levels was 6.74 mmol L^−1^ (standard deviation 2.73). The average levels of low‐density lipoprotein cholesterol (LDLC) were 2.58 mmol L^−1^ (standard deviation 0.93). The mean level of high‐sensitivity C‐reactive protein (hs‐CRP) was 8.33 mg L^−1^ (standard deviation 17.18) (Table S1, Supporting Information). To be noted, correlation and regression analyses indicated a significant positive correlation between PA and hs‐CRP (estimate = 6.24, P = 0.001), which is well accepted as a key inflammatory marker and strong independent predictor of cardiovascular risk^[^ ^14^ ^]^ (**Figure** **1**A). The mediating effects of hs‐CRP on the association between PA and death or MACE were further explored by the mediation analysis. As shown in Table S2 (Supporting Information), hs‐CRP mediated the relationship between PA and the risk of mortality in CHD patients to a certain extent, while no significant mediation effect was found for MACE. In addition, biomarkers associated with CVD, including GLUC, APOB (Apolipoprotein B), NEUT1 (neuter), and CHOL (cholesterol), showed a significant positive correlation with plasma PA level (Figure 1B–E). Interestingly, the result of the restricted cubic spline (RCS) showed that the hazard ratio (HR) of PA to death was higher at both low and high levels of PA than at the moderate level of PA, thus indicating that the association between PA concentration and the risk of mortality was nonlinear (Figure 1F). We tried to determine the cutpoint of PA level, using the maximum rank statistical method to obtain a significant grouping node. As indicated by Figure 1G,H, plotting the Kaplan‐Meier (KM) survival curve, we found that patients in the high PA level group (> standardized value 0.29) had lower survival rates (p = 0.033). At the same cutpoint of PA level (0.29), the risk of MACE was significantly higher in the high PA group compared to the low PA group (p = 0.013) (Figure 1I). Therefore, these clinical data provide evidence that high level of PA was associated with risk of death and MACE in CHD patients, and that cardiovascular risk were significantly increased when the PA level was over cutpoint 0.29.

### 2.2 PA Accelerated Cardiovascular Dysfunction and Endothelial Cell Injury In Vivo and In Vitro

**Figure 2:**
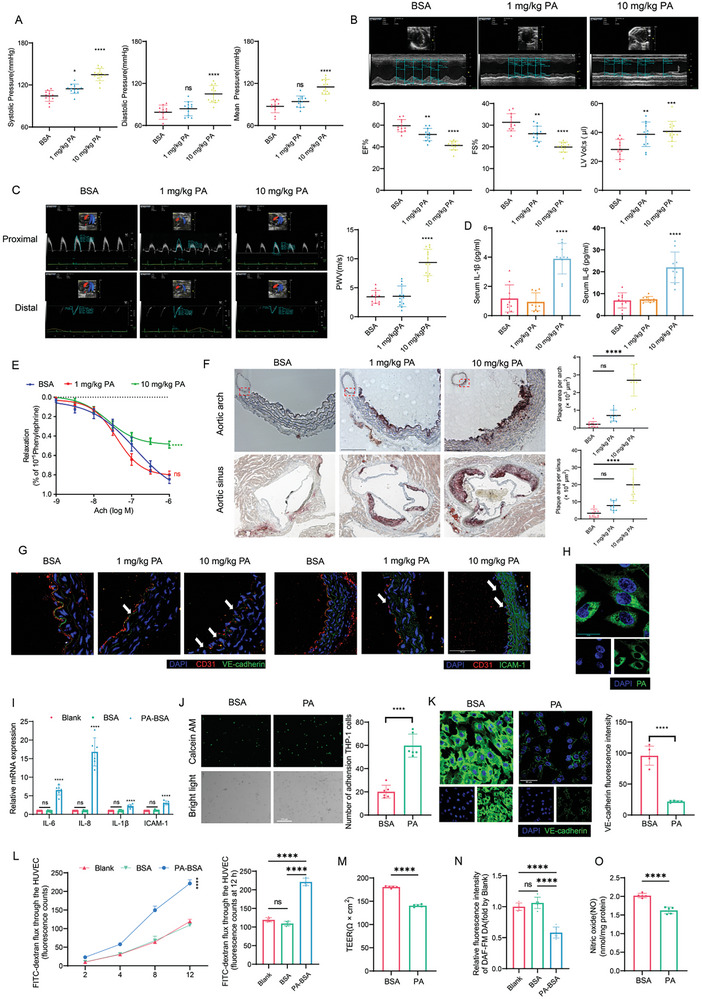
PA induced cardiovascular dysfunction of ApoE^−/‐^ mice and HUVECs inflammation. A‐G) ApoE^−/‐^ mice aged 6 weeks were divided into three groups and intraperitoneally administrated with BSA or 1 mg kg^−1^ PA or 10 mg kg^−1^ PA individually for 10 weeks. (A) Mice systolic, diastolic, and mean blood pressure were measured by noninvasive blood pressure monitor after administration (n = 11). (B and C) Statistic results and representative images of echocardiographic measurements of fractional shortening (FS), ejection fraction (EF), left ventricular end‐systolic volume (LV vol; s) and pulse wave velocity (PWV) (n = 12). Scale bar, 100 ms (horizontal) and 200 mm (vertical). (D) The concentration of IL‐6 and IL‐1β in serum were measured by ELISA (n = 10). (E) The vasodilation responses to different concentration of acetylcholine (ACh) after treating phenylephrine (PE) (n = 6). (F) The respective images and quantitative plaque area of oil red O staining of aortic sinus (n = 11). Scale bars, 250 µm. (G) The respective images of immunofluorescence of aortic arch sections stained by CD31 (red, as an endothelial marker), VE‐cadherin (green, as endothelial barrier marker), or ICAM‐1 (green, as proinflammatory factor), and DAPI (blue). Arrowheads indicate CD31 or VE‐cadherin incontinuity (n = 6). Scale bars, 100 µm. H‐M) HUVECs were treated with control BSA or 500 µM PA‐BSA for 24 h. (H) Representative images of HUVECs which were treated with fluorescently marked PA (BODIPY® FL C16, green) and stained by PKM2 (red). Scale bars, 20 µm. (I) The relative mRNA level of inflammatory cytokines (*IL‐6*, *IL‐8*, and *IL‐1β*) and adhesion molecule (*ICAM‐1*) in HUVECs (n = 3). (J) Representative images and statistical result of THP‐1 adhesion to HUVECs. Calcein AM (5 µM)‐labeled THP‐1 cells were added into HUVECs for 2 h, and After removing the non‐adherent THP‐1 cells, the adherent THP‐1 cells with green fluorescence (calcein AM) and bright field were visualized and counted (n = 6). Scale bars, 275 µm. (K) Representative images and statistical result of immunofluorescence of HUVECs which were stained by VE‐cadherin (green) and DAPI (blue) (n = 5). Scale bars, 50 µm. (L) 1 mg/mL FITC‐dextran was added to upper chamber of transwell plated HUVECs and the fluorescence intensity of lower chamber was measured along with time (n = 5). (M) The TEER value was calculated by (Experimental Resistance – Blank Resistance) × pore area (n = 5). N) Relative NO level of HUVECs (fold by Blank). DAF‐FM DA reacted with NO to excess NO level (n = 5). O) NO content was measured by NO Griess method kit (n = 5). Statistical significance was calculated using two‐sided Student's t‐tests or one‐way ANOVA. Data are shown as mean ±SD. *P < 0.05; **P < 0.01; ***P < 0.001; ****P < 0.0001. ns means no significance.

To confirm our clinical findings, the deterioration effect of PA on cardiovascular system was studied in 6‐week‐old ApoE^−/‐^ mice that were intraperitoneally administrated with bovine serum albumin (BSA)‐conjugated PA for 10 weeks. In consideration of the concentration of PA in human blood^[^ ^15^, ^16^, ^17^ ^]^ and its absorption, 1 mg kg^−1^ or 10 mg kg^−1^ PA was applied. There was no difference in body weight between groups (Figure S1a, Supporting Information). Importantly, we found that 10 mg kg^−1^ PA significantly increased the systolic, diastolic and mean blood pressure (**Figure** **2**A) Echocardiography analysis showed left ventricular (LV) fractional shortening (FS) and ejection fraction (EF) were reduced but left ventricular end‐systolic volume (LV vol; s) was increased in 10 mg kg^−1^ PA‐treated mice (Figure 2B), without alteration in left ventricular end‐diastolic volume (LV vol; d) or left ventricular posterior wall (LVPW) (Figure S1b, Supporting Information), indicating that high level of PA could induce LV contractile dysfunction and remodeling. Besides, pulse wave velocity (PWV) of aortic arch was significantly increased in 10 mg kg^−1^ PA group, suggesting that PA facilitates vascular sclerosis (Figure 2C). Moreover, the serum levels of inflammatory cytokines interleukin IL‐6 and IL‐1β were elevated by high concentration of PA treatment (Figure 2D). To further investigate the effect of PA on the vascular function, the thoracic aortas of mice were dissected for isometric tension recording. As implied by the results, endothelium‐dependent relaxations induced by acetylcholine were remarkably impaired by 35.8% in the aortas of 10 mg kg^−1^ PA‐treated mice, as compared to the control mice (Figure 2E). Oil red O staining results demonstrated high lipid deposition in the aortic arch and aortic sinus of mice treated with PA (Figure 2F). Co‐staining with CD31 (endothelial marker) and VE‐cadherin (endothelial cell adherence junction marker) in aortic arch sections exhibited that vascular endothelial integrity was impaired and vascular permeability was disrupted by PA administration (Figure 2G). The expression of intercellular cell adhesion molecule‐1 (ICAM‐1), an endothelial‐leukocyte adhesion molecule,^[^ ^18^, ^19^ ^]^ was increased by PA (Figure 2G). Accordingly, these in vivo findings indicate that elevated concentrations of PA might trigger cardiovascular dysfunction.

In vitro, the detrimental effect of PA was investigated in ECs. We first used fluorescently labeled PA to confirm that PA could passed through cytomembrane and was accumulated in cytosol (Figure 2H). Next, human umbilical vein endothelial cells (HUVECs) were challenged with 500 µM PA‐BSA for 24 h. Quantitative PCR results showed the levels of inflammatory cytokines including *IL‐6*, *IL‐8*, and *IL‐1β*, as well as the adhesion molecule *ICAM‐1*, were significantly higher in the PA‐treated group than in the control group (Figure 2I). Since recruitment of monocytes to the endothelium is a hallmark for endothelial activation and vascular inflammation,^[^ ^19^ ^]^ THP‐1 monocytes adhesion assay was conducted. As shown in Figure 2J, the number of THP‐1 cells recruited and adhered to HUVECs was significantly augmented by PA. Immunofluorescence showed that PA reduced the expression of endothelial barrier protein VE‐cadherin, implying an impairment of endothelial barrier (Figure 2K). Endothelial cell permeability was measured by the passage of FITC‐labeled dextran across monolayers of HUVECs and by trans‐endothelial electrical resistance (TEER). The results demonstrated that PA promoted more FITC‐dextran passed through HUVECs along with time, and that PA significantly decreased TEER value, thus confirming that PA could disrupt endothelial permeability and reduce endothelial barrier integrity (Figure 2L–M). Furthermore, nitric oxide (NO) production detected by DAF‐FM DA and Griess assay probe indicated that PA repressed NO level (Figure 2N–O). The influence of BSA to endothelial inflammation, permeability, and NO production was excluded as well (Figure 2I,L,N). Taken together, consistent with our in vivo observations, these data indicate that PA facilitates endothelial injury and impairs endothelial barrier function, probably contributing to cardiovascular dysfunction.

### 2.3 Protein Palmitoylation was Involved in PA‐Induced Endothelial Cell Injury

**Figure 3:**
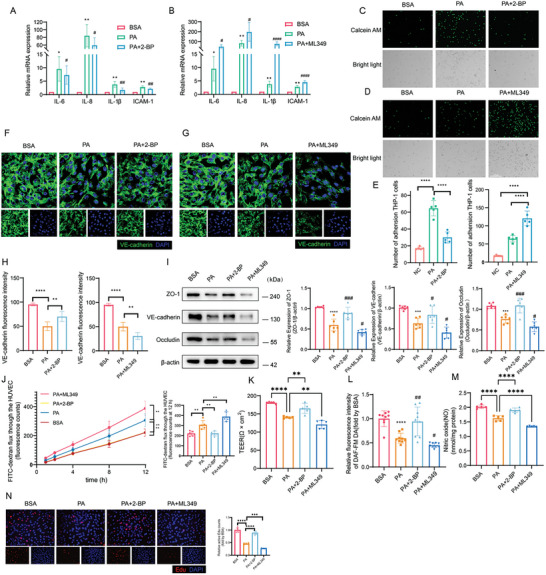
Endothelial palmitoylation regulated PA‐induced inflammation. HUVECs were treated with control BSA or 500 µM PA‐BSA for 24 h, besides 5 µM 2‐BP for 10 h or 8 µM ML349 for 48 h. A,B) The relative mRNA expression of *IL‐6*, *IL‐8*,*IL‐1β*, and *ICAM‐1* (n = 5). C‐E) Representative images and statistical result of THP‐1 adhesion to HUVECs. The adherent THP‐1 cells with green fluorescence(5 µM calcein AM) and bright field were visualized and counted (n = 5). Scale bars, 275 µm. F‐H) Representative images and statistical result of immunofluorescence of HUVECs which were stained by VE‐cadherin (green) and DAPI (blue) (n = 6). Scale bar, 50 µm. I) Representative immunoblots and statistical results of ZO‐1, VE‐cadherin and Occludin (n = 6). J) The fluorescence intensity of lower transwell chamber was measured along with time (n = 5). K) The TEER value was calculated by (Experimental Resistance – Blank Resistance) × pore area (n = 5). L) Relative NO level of HUVECs (fold by BSA). DAF‐FM DA reacted with NO to excess NO level (n = 9). M) NO content was measured by NO Griess method kit (n = 5). (N) EdU staining (red) showing the ratio of proliferating endothelial cells. scale bar: 125 µm or 200 µm (n = 5). Statistical significance was calculated using one‐way ANOVA. Data are shown as mean ±SD. *P < 0.05; **P < 0.01; ***P < 0.001; ****P < 0.0001; # versus PA.

Since PA may serve as the substrate for protein palmitoylation, it was investigated whether or not PA induces endothelial dysfunction via palmitoylation. Pharmacological inhibitors were used to manipulate protein palmitoylation, including 2‐bromopalmiate (2‐BP) as a global inhibitor of palmitoyl‐transferases, as well as ML348 and ML349 for selective inhibition of depalmitoylases APT1 and APT2 respectively. The results showed that 2‐BP attenuated PA‐induced upregulation of inflammatory cytokines and adhesion molecules (**Figure** **3**A), prevented THP‐1 cells adhesion to HUVECs (Figure 3C,E), augmented expressions of VE‐cadherin (Figure 3F,H), Occludin and Zona occludens‐1 (ZO‐1) (Figure 3I), improved endothelial cell membrane permeability (Figure 3J‐K), enhanced NO production (Figure 3L‐M), and facilitated endothelial proliferation (Figure 3N) These observations thus suggest that inhibition of protein palmitoylation might reverse PA‐mediated endothelial inflammation and impairment of endothelial barrier function. In contrast, ML349 but not ML348 aggravated endothelial dysfunction induced by PA (Figure 3; Figure S2, Supporting Information), indicating that promotion of protein palmitoylation by APT2 inhibition could further exacerbate the detrimental effect of PA. Taken together, these data prompt the conclusion that protein palmitoylation contributes to impairment of endothelial function by PA.

### 2.4 PKM2 Palmitoylation at cys31 was Required for PA‐Induced Endothelial Cell Injury

**Figure 4:**
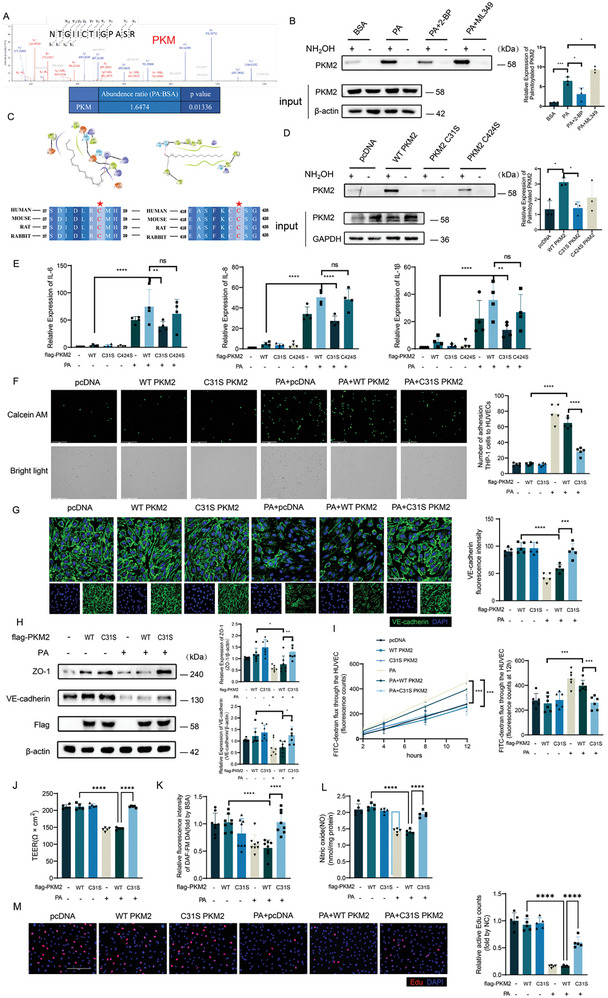
PKM2 C31 site palmitoylation regulated endothelial cell injury. A) Peptide mass fingerprinting of palmitoylated PKM from MS analysis. B) Representative immunoblots of Acyl‐RAC in HUVECs with PA, 2‐BP, or ML349 treatments (n = 3). C) Molecular docking predicted palmitoyl group might be incorporated with PKM2 C31 or C424 two sites respectively. The table showed highly conserved sequence of both sites and red stars indicate the cysteine residual. (D‐J) HUVECs transiently transfected with plasmids of WT, C31S, or C424S PKM2‐Flag. D) Representative immunoblots of palmitoylated and input PKM2 (n = 3). (E) Relative mRNA level of *IL‐6*, *IL‐8*, and *IL‐1β* (n = 4). F) Representative images and statistical result of THP‐1 adhesion to HUVECs. The adherent THP‐1 cells stained by 5 µM calcein AM were visualized and counted (n = 5). Scale bars, 275 µm. G) Representative images and statistical result of immunofluorescence of HUVECs which were stained by VE‐cadherin (green) and DAPI (blue) (n = 5). Scale bars, 50 µm. H) Representative immunoblots and statistical result of ZO‐1, VE‐cadherin, and Flag (n = 7). I) The fluorescence intensity of lower transwell chamber was measured along with time (n = 6). J) The TEER value was calculated by (Experimental Resistance – Blank Resistance) × pore area (n = 5). K) Relative NO level of HUVECs. DAF‐FM DA reacted with NO to excess NO level (n = 8). L) NO content was measured by NO Griess method kit (n = 5). M) EdU staining (red) shows the ratio of proliferating endothelial cells. scale bar: 125 µm or 200 µm (n = 5). Statistical significance was calculated using one‐way ANOVA. Data are shown as mean ±SD. *P < 0.05; **P < 0.01; ***P < 0.001; ****P < 0.0001. ns means no significance.

To explore underlying mechanisms by which palmitoylation regulates endothelial cell injury, potential palmitoylated proteins in ECs were identified by palmitoyl‐proteomics analysis through Acyl resin‐assisted capture (Acyl‐RAC) assay. As a result, 319 palmitoylated proteins were identified and KEGG analysis revealed that 40 proteins among them were involved in metabolic pathways (Figure S3a,b, Supporting Information). Interestingly, PKM, the key rate‐limiting enzyme of glycolysis, was observed with 1.65 fold increase of palmitoylation level in PA‐treated ECs as compared to control (**Figure** **4**A; Figure S3b, Supporting Information). Considering that PKM2 is the predominant‐expressed PKM subtype in ECs,^[^ ^20^ ^]^ it was speculated that PKM2 was the palmitoylated substrate protein involved in PA‐induced endothelial dysfunction. Indeed, the Acyl‐RAC assay data confirmed that palmitoylation of PKM2 was induced by PA, which was reversed by 2‐BP but was further enhanced by ML349 (Figure 4B).

Next, bioinformatic analysis with CSS‐Palm 4.0 and molecular docking were performed to predict the potential cysteine sites for PKM2 palmitoylation. Only Cys31 and Cys424, among the total 10 cysteine sites for assessment, were predicted to have the highest potential for palmitoylation with highly conserved sequence (Figure 4C; Figure S3c, Supporting Information). To confirm these palmitoylation sites, we employed C31S and C424S mutants (cysteine replaced by serine) in Acyl‐RAC assays. We found that C31S mutant drastically inhibited palmitoylation signals compared to wildtype PKM2 or C424S mutant (Figure 4D), implying that PKM2 is mainly palmitoylated at Cys31.

To elucidate whether or not PKM2 palmitoylation at Cys31 participates in PA‐induced endothelial cell dysfunction, C31S mutant was transfected into HUVECs followed by treatment of PA. As shown in Figure 4E–M, C31S mutant significantly reversed PA‐induced endothelial inflammation and endothelial barrier disruption as compared to wildtype PKM2, based on the observations that C31S diminished the expressions of inflammatory cytokines, prevented THP‐1 cells adhesion to ECs, recovered expressions of endothelial junctional proteins, repaired endothelial permeability and NO production, as well as improved cell proliferation. By contrast, C424S mutant was not able to change PA‐induced endothelial inflammation (Figure 4E). Therefore, these findings support the view that PKM2 palmitoylation at cys31 plays a pivotal role in PA‐induced endothelial dysfunction.

### 2.5 Ablation of Endothelial PKM2‐C31 Palmitoylation Mitigated PA‐Induced Cardiovascular Dysfunction

**Figure 5:**
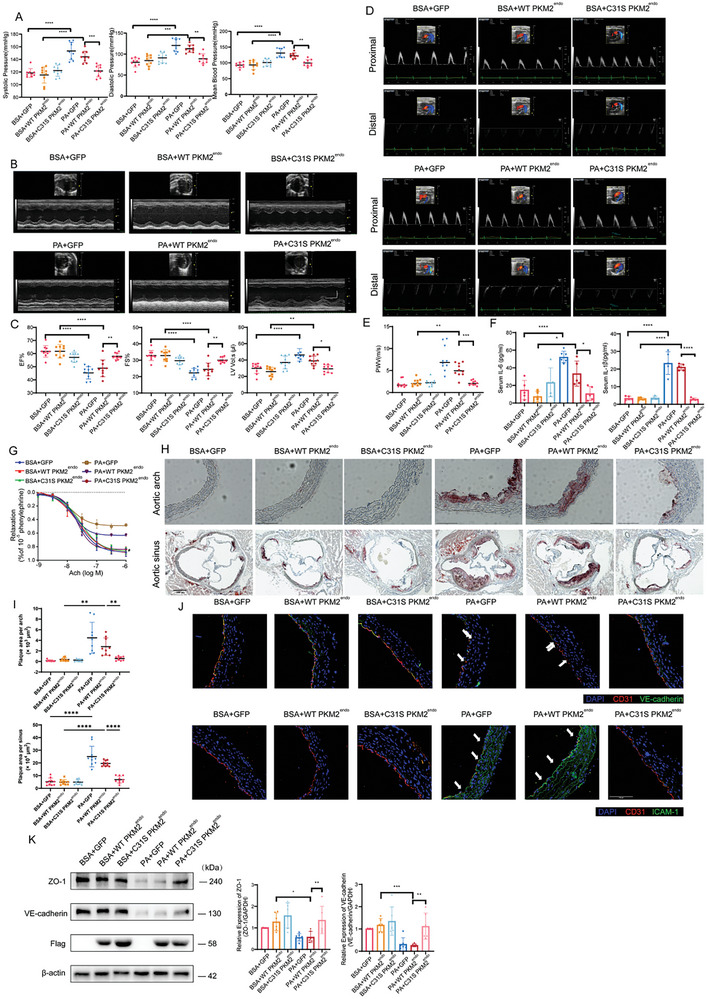
Vascular endothelial C31S PKM2 mitigated PA‐induced cardiovascular dysfunction ApoE^−/‐^ mice aged 6 weeks were divided into six groups and intraperitoneally administrated with BSA or 10 mg kg^−1^ PA individually for 10 weeks as well as tail intravenously injected with endothelial specifically expressed WT or C31S PKM2 once every five weeks. A) Mice systolic, diastolic, and mean blood pressure were measured by noninvasive blood pressure monitor after administration(n = 9). B‐E) Statistic results and representative images of echocardiographic measurements of fractional shortening (FS), ejection fraction (EF), left ventricular end‐systolic volume (LV vol; s) and pulse wave velocity (PWV) (n = 10). Scale bar, 100 ms (horizontal) and 200 mm (vertical). F) The concentration of IL‐6 and IL‐1β in serum were measured by ELISA (n = 5). G) The vasodilation responses to different concentration of acetylcholine (ACh) after treating phenylephrine (PE) (n = 6). H,I) The respective images and quantitative plaque area of oil red O staining of aortic sinus (n = 10). Scale bars, 250 µm. J) The respective images of immunofluorescence of aortic arch sections stained by CD31 (red, as an endothelial marker), VE‐cadherin (green, as endothelial barrier marker), or ICAM‐1(green, as proinflammatory factor), and DAPI (blue). Arrowheads indicate CD31 or VE‐cadherin incontinuity (n = 6). Scale bars, 100 µm. K) Representative immunoblots and statistical results of ZO‐1 and VE‐cadherin (n = 6). Statistical significance was calculated using two‐sided Student's t‐tests or one‐way ANOVA. Data are shown as mean ±SD. *P < 0.05; **P < 0.01; ***P < 0.001; ****P < 0.0001.

To further confirm whether PKM2‐C31 palmitoylation in ECs contributes to PA‐induced cardiovascular dysfunction, we performed in vivo experiments by intravenous injection of endothelial‐specific ‐adeno‐associated virus (AAV‐C31S PKM2^endo^ and AAV‐WT PKM2^endo^) in ApoE^−/‐^ mice receiving with or without PA treatment (10 mg kg^−1^, for 10 weeks). Here, we observed no body weight change within the groups (Figure S4a, Supporting Information). Immunofluorescence staining of aortic endothelium revealed that those AAVs successfully induced the exogenous expression of WT and mutant PKM2 in ECs (Figure S4b, Supporting Information). Compared with WT PKM2^endo^, C31S PKM2^endo^ decreased blood pressure (**Figure** **5**A), improved myocardial systolic function, and ameliorated cardiac remodeling (Figure 5B,C; Figure S4c, Supporting Information), mitigated stiffness of aortic arch (Figure 5D,E), diminished serum IL‐6 and IL‐1β in PA‐treated ApoE^−/‐^ mice (Figure 5F). Isometric tension assay indicated that C31S PKM2^endo^ rescued the blunted endothelial‐dependent relaxations caused by PA administration (Figure 5G). In addition, C31S PKM2^endo^ inhibited atherosclerotic plaque formation at aortic arch and aortic sinus, based on Oil red O staining (Figure 5H–I). Immunofluorescence experiments revealed that C31S PKM2^endo^ restored endothelial marker CD31 and barrier protein VE‐cadherin, but decreased expression of adhesion molecule ICAM‐1 in aortic arch (Figure 5J). Besides, C31S PKM2^endo^ rebounded the expressions of ZO‐1 and VE‐cadherin in aorta (Figure 5K). Taken together, these data indicate that PKM2‐C31 palmitoylation participates in PA‐induced cardiovascular dysfunction, ablation of PKM2 palmitoylation at Cys31 in ECs improves cardiovascular homeostasis.

### 2.6 PKM2‐C31 Palmitoylation Facilitated PA‐Induced Endothelial Dysfunction through Suppressing PKM Activity and Endothelial Glycolysis

**Figure 6:**
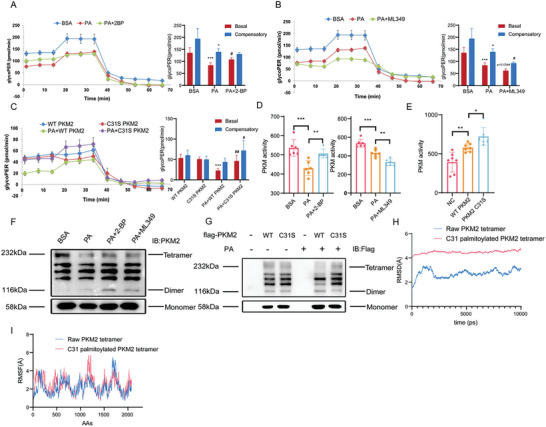
PKM2 C31S mutant ameliorated inflammation by improving glycolysis in HUVECs. A‐C) The Seahorse XFe96 Extracellular Analyzer was applied to record glycolytic proton efflux rate (glycopPER), followed by injected with Rotenone/Antimycin (Rot/AA) and 2‐deoxy‐D‐glucose (2‐DG) according to the manufacturer's protocol. Data were automatically generated by Wave program and Seahorse XF Glycolytic Rate Assay Report Generator (n = 5). D‐E) Statistical results of PKM activity measured by corresponding kit (n = 6 or 7). F‐G) Representative immunoblots of nondenaturing gel electrophoresis to assess of monomeric, dimeric, and tetrameric PKM2 (n = 5 or 3). H‐I) RMSD and RMSF analyzed by molecular dynamics simulation to evaluate the stability of raw PKM2 tetramer (red) and C31 palmitoylated PKM2 tetramer (blue). Statistical significance was calculated using one‐way ANOVA. Data are shown as mean ±SD. *P < 0.05; **P < 0.01; ***P < 0.001; # versus PA.

We attempted to elucidate the mechanism of PKM2‐C31 palmitoylation leading to endothelial cell injury. Since ECs primarily rely on glycolysis as energy source,^[^ ^21^ ^]^ and since PKM2 is the key rate‐limiting enzyme of glycolysis, the hypothesis was tested that palmitoylation of PKM2 affects it enzymatic activity, ultimately regulates endothelial glycolysis and ATP production. First, glycolysis rate assay was performed to assess the change of glycolysis of HUVECs. PA significantly decreased glycolytic proton efflux rate (glycoPER), which was reversed by 2‐BP and was aggravated by ML349 (**Figure** **6**A,B). Compared to WT PKM2, C31S PKM2 improved PA‐induced decrease of glycoPER, indicating that PKM2‐C31 palmitoylation is involved in the impairment of endothelial glycolysis (Figure 6C). Second, PA facilitated a decline of PKM activity, which could be inhibited by 2‐BP or C31S PKM2, but rather exacerbated by ML349 (Figure 6D,E). Third, PA reduced ATP production and lactic acid level in ECs, whereas these effects were suppressed by 2‐BP and potentiated by ML349 (Figure S5, Supporting Information). Consistent with these observations, activation of PKM2 by TEPP‐46 attenuated PA‐induced production of inflammatory cytokines, while inhibition of endothelial glycolysis by 3‐BP (a hexokinase II inhibitor) or PFK15 (a 6‐phosphofructo‐2‐kinase inhibitor) enhanced PA‐induced endothelial inflammation (Figure S6, Supporting Information), further supporting the view that glycolysis plays a key role in maintaining endothelial cell function.

It was unclear how PKM2‐C31 palmitoylation represses its enzymatic activity. We first investigated whether PKM2‐C31 palmitoylation influence the formation of PKM2 tetramer, which has been reported to be important for PKM activity.^[^ ^22^ ^]^ By using nondenaturing gel electrophoresis, the tetrameric form of PKM2 was observed to be reduced by PA, but the tetramer was preserved in the presence of 2‐BP and C31S (Figure 6F,G). Molecular dynamics simulation showed that PKM2‐C31 palmitoylation with higher RMSD and RMSF impaired the stability of PKM2 tetramer (Figure 6H,I). We also tested the possibility that PKM2‐C31 palmitoylation might facilitate its nuclear translocation, since PKM2 could translocate to the nucleus to activate gene transcription resulting in inflammatory response.^[^ ^23^ ^]^ However, neither PA, 2‐BP nor ML349 affected PKM2 nuclear translocation (Figure S7, Supporting Information), thus excluding this possibility.

Taken in conjunction, these results strongly suggest that PKM2‐C31 palmitoylation impairs endothelial function via blocking its tetramerization and inhibiting its enzyme activity, finally suppressing glycolysis in ECs.

### 2.7 PKM2 was Palmitoylated by zDHHC13

**Figure 7:**
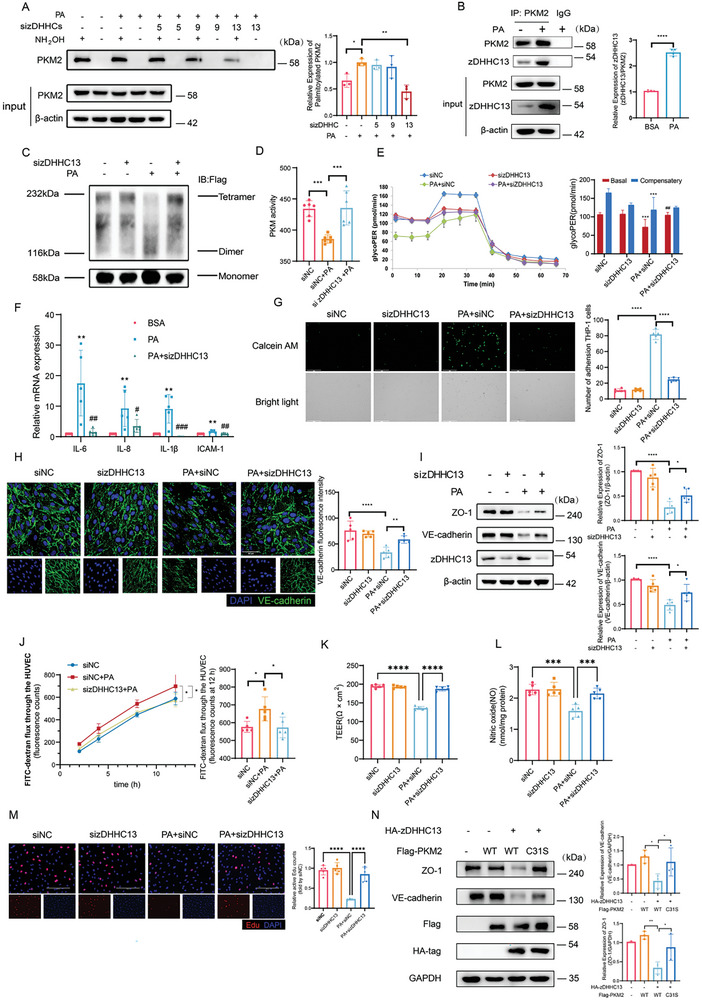
zDHHC13 palmitoylated PKM2 leading to endothelial injury via inhibition of glycolysis. A) Acyl‐RAC assay of HUVECs after silencing zDHHC5, 9 and 13 (n = 3). B) Representative images of immunoprecipitation of PKM2 in PA‐treated HUVECs (n = 4). C‐J) zDHHC13 knockdown HUVECs were challenged with 500 µM PA. (C) Representative immunoblots of nondenaturing gel electrophoresis to assess PKM2 monomeric, dimeric, and tetrameric (n = 3). (D) Statistical analysis of PKM activity measured by corresponding kit (n = 6). (E) The Seahorse XFe96 Extracellular Analyzer was applied to record glycolytic proton efflux rate (glycopPER), followed by injected with Rotenone/Antimycin (Rot/AA) and 2‐deoxy‐D‐glucose (2‐DG) according to the manufacturer's protocol. Data were automatically generated by Wave program and Seahorse XF Glycolytic Rate Assay Report Generator (n = 5). (F) Relative mRNA level of *IL‐6*, *IL‐8, IL‐1β* and *ICAM‐1* (n = 5). (G) Representative images and statistical result of THP‐1 adhesion to HUVECs. The adherent THP‐1 cells stained by 5 µM calcein AM were visualized and counted (n = 5). Scale bars, 275 µm. (H) Representative images and statistical result of immunofluorescence of HUVECs which were stained by VE‐cadherin (green) and DAPI (blue) (n = 5). Scale bars, 50 µm. (I) Representative immunoblots and statistical result of ZO‐1, VE‐cadherin, and zDHHC13 (n = 5). (J) The fluorescence intensity of lower transwell chamber was measured along with time and at 12 hours (n = 5). K) The TEER value was caculated by (Experimental Resistance – Blank Resistance) × pore area (n = 5). L) NO content was measured by NO Griess method kit (n = 5). M) EdU staining (red) shows the ratio of proliferating endothelial cells. scale bar: 125 µm or 200 µm (n = 5). N) HUVECs were overexpressed with HA‐zDHHC13 and Flag‐WT/C31S PKM2. Representative immunoblots of ZO‐1, VE‐cadherin, HA, and Flag (n = 3). Statistical significance was calculated using one‐way ANOVA. Data are shown as mean ±SD. *P < 0.05; **P < 0.01; ***P < 0.001; ****P < 0.0001; # versus PA.

To find out the palmitoyl‐transferases responsible for PKM2 palmitoylation, we selected 11 out of 23 total zDHHCs, which are confirmed to be highly expressed in ECs,^[^ ^24^ ^]^ as candidates for investigation. First, we tested the mRNA expressions of 11 zDHHCs in PA‐treated HUVECs. As shown, zDHHC5, zDHHC9, and zDHHC13 were increased in response to PA (Figure S8a, Supporting Information). Next, we analyzed a GEO dataset which studied genome‐wide expression of adjacent artery/carotid plaque from hypertensive patients. Consistent with our qPCR results, zDHHC5, zDHHC9, and zDHHC13 were high‐expressed in aortic plaque (Figure S8b, Supporting Information), suggesting that these three zDHHCs might be probably involved in PA‐induced inflammation. We silenced these zDHHCs (Figure S8c, Supporting Information) and performed Acyl‐RAC assay, and found that only zDHHC13 silencing could decrease PKM2 palmitoylation (**Figure** **7**A). Besides, co‐immunoprecipitation and immunofluorescence results confirmed that there was interaction between PKM2 and zDHHC13 (Figure 7B; Figure S8d, Supporting Information).

To further evaluate the potential of zDHHC13 as a candidate responsible for PKM2 palmitoylation, PKM2 tetramer formation, and PKM activity, glycolysis, as well as endothelial functions were investigated following zDHHC13 silencing. Knockdown of zDHHC13 increased PKM2 tetramer (Figure 7C), restored PKM activity (Figure 7D), and improved glycoPER (Figure 7E) in PA treatment group. Additionally, silencing of zDHHC13 decreased mRNA expressions of inflammatory cytokines and adhesion molecules (Figure 7F), attenuated THP‐1 adhesion (Figure 7G), enhanced expressions of VE‐cadherin and ZO‐1 (Figure 7H,I), improved endothelial barrier function (Figure 7J,K), increased endothelial NO content (Figure 7L) and enhanced proliferation property (Figure 7M). By the contrast, overexpression of zDHHC13 reduced endothelial barrier function (Figure 7N). Most importantly, the effect of zDHHC13 overexpression could be reversed by C31S PKM2 (Figure 7N). Taken together, these data indicate that zDHHC13 palmitoylates PKM2 and accelerates PA‐induced endothelial dysfunction.

## 3 Discussion

Accumulating epidemiological evidences demonstrate that high serum level of PA, no matter by excessive dietary intake or by aberrant plasma FA profile, is associated with the incidence and severity of cardiovascular diseases including CHD.^[^ ^2^, ^5^, ^6^ ^]^ In the present study, we provide novel insight into the role of abnormal PA accumulation in risk of death and MACE events in CHD patients by analyzing the clinical cohort data of 1040 patients. Our study confirms that PA may serve as a potential biomarker for cardiovascular risk. This conclusion is supported by the following results: (1) plasma concentration of PA was associated with the risk of mortality in a non‐linear manner, wherein PA level exceed the optimal range would increase the mortality rate of CHD; (2) patients with higher PA levels have higher risk of MACE and death, but lower survival rates, as compared to those with lower PA levels. Additionally, PA level was positively correlated to the inflammatory biomarkers hs‐CRP and NEUT1, as well as biomarkers of metabolic dysfunction GLUC, APOB and CHOL, suggesting that CHD patients with higher PA levels are accompanied with cardiovascular inflammatory and abnormal glucose and lipid metabolism. Notably, vascular inflammation has been proved to be a strong driver of cardiovascular risk, and hs‐CRP has been identified as a pivotal predictor for risk of adverse cardiovascular events and death.^[^ ^25^ ^]^ The mediation analysis indicated that the indirect effect of PA on death, mediated through hs‐CRP, was statistically significant, thus supporting the mediating effects of hs‐CRP on the association between PA and risk of mortality in CHD patients. Therefore, the positive correlation between PA and hs‐CRP in CHD patients strongly suggests that PA might mediate inflammation in the cardiovascular system to increase subsequent cardiovascular event risk.

In line with these clinical studies, excess intake of PA in the dyslipidemia ApoE^−/‐^ mice elevated blood pressure, impaired cardiac contractile function, induced cardiac remodeling and vascular sclerosis. These observations are consistent with a recently published study that PA accumulation markedly increased plaque burden and triggered cardiovascular risks in patients with Type 2 Diabetes Mellitus.^[^ ^26^ ^]^ Interestingly, the detrimental role of PA in the cardiovascular system was only observed in the ApoE^−/−^ mice, but not in the normal control C57BL/6 mice (Figure S9, Supporting Information). It is suspected that PA is eliminated better and is not accumulated in the normal body. Only if PA is accumulated to a certain level in the body, PA might cause cardiovascular injury. This speculation is supported by our results that PA at the given dose of 10 mg kg^−1^, but not 1 mg kg^−1^, could induce cardiovascular injury. In particular, our data revealed that PA remarkably induced endothelial injury and dysfunction, as implied by the increased secretion of inflammatory cytokines and adhesion molecules, monocyte recruitment and lipid deposition in the vasculature, the impaired endothelium‐dependent relaxations and reduced production of NO, as well as the disrupted endothelial permeability and barrier integrity. In view of the vital role of endothelial inflammation and dysfunction in the development of CVD,^[^ ^27^ ^]^ these in vivo and in vitro results thus imply that the cardiovascular deterioration by PA is probably attributed to endothelial cell injury.

Most importantly, our findings indicate that palmitoylation is involved in PA‐induced endothelial cell injury. This conclusion is supported by the observations that inhibition of palmitoylation by palmitoyl‐transferase inhibitor 2‐BP could eliminate PA‐induced inflammation and dysfunction in ECs, whereas promotion of palmitoylation by depalmitoylase APT2 inhibitor ML349 exacerbated the harmful effect of PA. These findings emphasize the importance of palmitoylation as a novel mechanism underlying the cardiovascular impairment by PA, beyond its well‐known lipotoxic and oxidative properties.^[^ ^28^ ^]^ Indeed, since PA provides substrates for protein palmitoylation, the elevated circulating PA might enhance protein palmitoylation. As a result, the palmitoylated proteins undergo changes in their structure, biological functions, or subcellular localization, thereby initiating pathophysiological events.^[^ ^29^, ^30^, ^31^ ^]^ Currently, a variety of palmitoylated proteins have been identified. Protein palmitoylation has been reported to be involved in the regulation of insulin resistance, myocardial ischemia‐reperfusion injury, and other CVD.^[^ ^32^, ^33^ ^]^

By palmitoyl‐proteomics analysis, PKM2 was identified as the palmitoylated protein responsible for PA‐induced endothelial cell injury. Based on Acyl‐RAC assay and molecular docking results, Cys31 was found to be the predominant palmitoylated site of PKM2. C31S mutant was employed to abolish PKM2‐C31 palmitoylation. The observations that C31S mutant prevented PA‐induced inflammation, barrier disruption, NO deficiency, and proliferation suppression in ECs, and that endothelial‐specific C31S mutation reversed the increased blood pressure, reduced cardiac contractile function, and atherosclerotic plaque formation caused by PA treatment in ApoE^−/‐^ mice, thus prompt the conclusion that PKM2‐C31 palmitoylation plays a key role in PA‐mediated cardiovascular dysfunction.

Taken into considerations that PKM2 catalyzes the last step of glycolysis by converting phosphoenolpyruvate into pyruvate,^[^ ^34^ ^]^ it is speculated that PKM2 palmitoylation at Cys31 may affect its pyruvate kinase activity and influence glycolysis in ECs. Indeed, the enzymatic activity of PKM2 and endothelial cell glycolysis rate were rebounded by C31S mutant or 2‐BP, but were worsened by ML349, supporting the view that PA‐mediated PKM2 palmitoylation is associated with an impairment of glycolysis in ECs. Differ from most somatic cells that mainly rely on oxidative phosphorylation for energy supply, ECs are addictive to glycolysis, since 85% of total ATP is produced by converting glucose to lactate. As a result, the pathophysiological role of endothelial glycolysis has received growing attention recently. Endothelial glycolysis modulates vessel sprouting and angiogenesis,^[^ ^35^, ^36^, ^37^ ^]^ endothelial permeability and vascular leakage,^[^ ^38^ ^]^ endothelial proliferation and senescence,^[^ ^39^ ^]^ inflammation and endothelial activation,^[^ ^40^ ^]^ as well as atherosclerosis progression.^[^ ^41^ ^]^ Although it remains controversial whether endothelial glycolysis plays a beneficial or detrimental role to maintain endothelial function, our study suggests that increased glycolysis in the ECs is a protective mechanism against PA‐induced injury. This is supported by the following observations: (1) PA reduced glycoPER in ECs; (2) inhibition of glycolysis by inhibitors of hexokinase II and 6‐phosphofructo‐2‐kinase aggravated PA‐induced endothelial inflammation; and (3) rescue of glycolysis by PKM2 activator ameliorated PA‐induced endothelial injury. Therefore, these data coincide with the observations that PKM2 C31S mutant enhanced endothelial glycolysis and conferred cardiovascular protection against PA, further convincing that the cardiovascular detrimental effect of PA‐mediated PKM2 palmitoylation is attributed to suppression of endothelial glycolysis.

Mechanistically, PKM2 palmitoylation repressed its activity via inhibiting its tetramer formation. PKM2 tetramer is essential for its enzymatic activity, whereas dissociation of PKM2 from tetramer into dimer is considered as a process of activity downregulation.^[^ ^42^ ^]^ PKM2 in tetramer state has an allosteric regulatory domain in its spatial structure like a seesaw. Allosteric regulators inserted into this spatial structure may alter the electrostatic force inside the molecule, finally promoting the dissociation.^[^ ^43^ ^]^ It is most likely that the insertion of palmitoyl group at Cys31 leads to change of electrostatic force. Our molecular dynamics simulation results that Cys31 palmitoylation destroyed the stability of PKM2 tetramer, may help to support this conclusion. Interestingly, although PKM2 shuttles to the nucleus to facilitate several pathological events,^[^ ^23^ ^]^ our data exclude the possibility that PKM2 palmitoylation affects its nuclear localization. Besides palmitoylation, PKM2 could also undergo post‐translational modifications (PTMs) such as phosphorylation,^[^ ^44^ ^]^ methylation,^[^ ^45^ ^]^ and O‐glycosylation,^[^ ^46^ ^]^ which might affect its enzymatic activity or nuclear translocation. However, our present findings do not permit further speculation on the crosstalk between palmitoylation and the other PTMs of PKM2, since the modification sites are quite different in these PTMs, and since Cys31 was not observed for other modifications.

Furthermore, our study suggests that PKM2‐C31 palmitoylation is regulated by zDHHC13 and APT2. Among the total 23 zDHHCs, zDHHC13 is highly expressed in ECs, and has been found to be upregulated in the aortic plaque of hypertensive patients and under PA stimuli. Knockdown of zDHHC13 repressed PKM2 palmitoylation and preserved tetramer formation, along with an improvement of glycolysis and endothelial function, whereas C31S mutant prevented endothelial injury caused by zDHHC13 overexpression. In addition to zDHHC13, palmitoylation of PKM2 is dynamically regulated by APT2, based on the observations that APT2 inhibitor ML349 increased PKM2‐C31 palmitoylation level, repressed PKM2 tetramerization and activity, impaired endothelial glycolysis, and aggravated endothelial cell injury. Our data also rule out the involvement of APT1, as implied by the results that APT1 inhibitor ML348 did not alter the effect of PA. This is probably due to their subcellular localization, since APT1 mediates depalmitoylation in mitochondria,^[^ ^47^ ^]^ while APT2 mainly resides in the cytosol and Golgi apparatus,^[^ ^48^, ^49^ ^]^ where it is nearby in space with the cytoplasm‐localized PKM2. Although de‐ palmitoylation is also catalyzed by PPTs and ABHDs, the present results do not permit further speculation on the involvement of PPTs and ABHDs in PKM2 de‐ palmitoylation.

As limitations of the present study, the PA levels were examined only on CHD patients, and the difference between CHD patients and healthy individuals was not able to be compared, since all the patients in this study came from a CHD cohort without healthy individuals. Additionally, the association between PA levels and CHD progression was not clarified in the follow‐up study. Further studies are needed to explore the relationship between PA levels and CVD incidence or development. Moreover, although our observations support speculation that PA causes cardiovascular harmful effects only when PA is accumulated to certain concentrations, the serum concentration of PA in mice after chronic treatment of PA was not measured. Furthermore, PKM2 knockout mice were not used to rule out the role of endogenous PKM2 before treatment of AAV‐C31S PKM2^endo^, due to the technical limitation of generating ApoE^−/−^ and PKM2^−/−^ double knockout mice.

In conclusion, the present study reveals that high level of PA is associated with cardiovascular risk, due to protein palmitoylation. Notably, S‐palmitoylation of PKM2 at cys31 accelerates endothelial inflammation and cardiovascular dysfunction. Mechanistically, palmitoylation of PKM2‐C31, which is regulated by zDHHC13 and APT2, inhibits endothelial glycolysis via repressing PKM2 tetramer formation. These data convince that PA may serve as a clinical biomarker for CVD, and suppression of PKM2 palmitoylation might have therapeutic potential against PA‐induced cardiovascular dysfunction.

## 4 Experimental Section

### Cohort Study

The clinical cohort studies adhered to the guidelines of the Helsinki Declaration and received ethics approval from the Medical Ethical Review Committee of Guangdong Provincial People's Hospital (GDREC2010137). The written consents were given prior to the inclusion of subjects in the study. This study is based on the previously published metabolomic study by Zhu et al.^[^ ^50^ ^]^ (Supplementary method). The methodology, conduct, and reporting of this study were in accordance with the Strengthening the Reporting of Observational Studies in Epidemiology (STROBE) Statement initiatives for cohort studies (Table S3, Supporting Information). The optimal cutoff value for palmitic acid (PA) associated with MACE was determined using the “survminer” R package, and this cutoff value classified patients into low PA and high PA groups. The relationship between PA levels and the occurrence of death and MACE was evaluated and compared using Kaplan‐Meier survival analysis and log‐rank tests.

### Palmitic Acid Preparation and Treatment

Since PA is insoluble in water, it is commonly conjugated to fatty acid‐free BSA to enhance its solubility as described.^[^ ^51^ ^]^ The stock solution was prepared by combination of PA (Aladdin) with fatty acid free BSA (Meilunbio). Briefly, 20% BSA was dissolved in ECM at 48 °C. PA was dissolved in 70 °C ethanol and was immediately added into 20% BSA to produce 2.5 mM PA solution, which were filter‐sterilized and stored at ‐20 °C. The control solution containing BSA and ethanol was prepared similarly.^[^ ^52^, ^53^, ^54^, ^55^ ^]^

### Experimental Animals

All animal experiments were conducted according to Institutional Animal Care and Use Committee of Sun Yat‐sen University (approval number: SYSU‐IACUC‐2023‐0112). The study conformed to the Guide for the Care and Use of Laboratory Animals published by the US National Institutes of Health (NIH Publication, 8th Edition, 2011). Six‐week‐old male ApoE^−/−^ mice were obtained from Zhuhai BesTest Bio‐Tech Co,.Ltd. and kept at a constant temperature (22 ± 1 °C) and 50% relative humidity under a 12 h/12 h light/dark cycle with free access to water and all fed with normal diet. According to literatures, the plasma concentration of PA in human range from 100 to 10 000 µmol L^−1^.^[^ ^15^, ^16^, ^17^ ^]^ Thus, mice were randomized into three groups and intraperitoneally injected with palmitic acid (PA‐BSA 1 mg kg^−1^ day^−1^ or 10 mg kg^−1^ day^−1^) or BSA (as control) for 10 weeks. Blood pressure and echocardiography were assessed before sacrifice. Mice were euthanized by administration of 45 mg kg^−1^ pentobarbital sodium.

For some experiments, AAV vectors cloned with endothelial‐specific promoter for intercellular adhesion molecule 2 (ICAM2) were applied to target endothelial cells. 6‐week‐old male ApoE^−/−^ mice were infected with the endothelial‐specific AAV GFP, AAV WT PKM2^endo^, or AAV C31S PKM2^endo^ through tail vein injection once every five weeks, followed by PA treatment (10 mg kg^−1^ day^−1^) for 10 weeks continuously. The recombinant adeno‐associated virus (AAV) was generated by Genechem Co.,Ltd. (Shanghai, China).

### Cell Culture and Treatment

HUVECs were isolated from human umbilical cords obtained from the First Affiliated Hospital, Sun Yat‐sen University, which were approved by Institutional Ethic Committee for Clinical Research and Animal Trials of the First Affiliated Hospital of Sun Yat‐sen University (approval number: [2020]051). The written consents were given prior to the inclusion of subjects in the study. THP‐1 cells were a gift from Prof. Huanhuan Liang (Sun Yat‐sen University). HUVECs were cultured in endothelial cell medium (ECM, ScienCell) supplemented with 5% FBS and 1% endothelial cell growth supplementary (ECGS), and were used within seven passages. THP‐1 cells were cultured in Roswell Park Memorial Institute (RPMI) 1640 (Gibco) with 10% FBS. All cells were cultured in an incubator with 5% CO_2_ at 37 °C. HUVECs were treated with 500 µM PA for 24 h. Simultaneously, cells were treated with 5 µM 2‐BP for 10 h, 8 µM ML348 or 8 µM ML349 for 48 h.

### Noninvasive Blood Pressure Measurement of Mice

Mice were calmed down and settled in temperature‐keeping restraining tubes. The tails of mice were ringed by pressure sensors. The systolic and diastolic pressure were measured by using noninvasive blood pressure measuring system, Coda 20 208 (Kent scientific corporation, USA) in real time.

### Echocardiography

Echocardiography in mice was performed as previously described.^[^ ^56^ ^]^ Mice were anesthetized in an airtight chamber with 5% isoflurane. Anesthesia mice were confirmed by disappeared pain reflex and were maintained with 1.5% isoflurane inhalation through a nasal cone. Vevo 2100 (Fuji VisualSonics, CAN) with a 38‐MHZ MS400 probe was used. The left ventricle was assessed in the parasternal short‐axis view and left ventricular (LV) fractional shortening (FS), ejection fraction (EF), and left ventricular end‐systolic volume (LV vol; s) were analyzed from M‐mode tracing. Pulse wave velocity (PWV) was analyzed from PW Doppler‐mode at aortic arch.

### Aortic Isometric Tension Recording Assay

As previous study described,^[^ ^57^ ^]^ the thoracic aortas from mice were dissected and immediately placed in Krebs‐Hensleit's Solution followed by removing connective tissue. Rings of thoracic aortas with 5 mm long were mounted in the isometric wire myogragh system, gassed with 5% CO_2_ and 95% O_2_. Before the treatment, aortic rings were slowly stretched to the optimal static tension of 3 mN and were allowed to equilibrate for 30 min. Aortic rings were challenged with 100 mM KCl to reach highest contraction. After three‐times washing, the aortic rings were contracted with 10 µM phenylephrine (PE, MCE), followed by cumulative addition of acetylcholine (ACh, 10^−9^ to 10^−6^ M). After that, the aortas were washed three times, and incubated with 0.1 mM L‐NAME (endothelial nitic oxide synthase inhibitor, Beyotime) for 15 min. PE and ACh were added into chamber as described above. Lastly, similarly incubated with L‐NAME and treated with PE, which followed by addition of 10^−10^ to 10^−5^ M sodium nitroprusside (SNP). Vasodilation responses were expressed as percentage of contractions to PE and recorded as cumulative concentration response curves.

### Plasmid and siRNA Transfection

Plasmids containing Flag‐PKM2‐WT, Flag‐PKM2‐C424S, Flag‐PKM2‐C31S, and HA‐zDHHC13 were obtained from Miaoling Bio (Wuhan, China). Small silencing RNAs (sizDHHC5 sizDHHC9 and sizDHHC13) were purchased from Ribo Bio (Guangzhou, China) and the sequences were listed in Table S5 (Supporting Information). Plasmids or siRNAs were transfected into 60%‐70% confluent HUVECs with Lipofectamine 2000 reagent (Invitrogen). Medium was changed to fresh ECM after 4–6  h transfection, and cells were maintained or treated with PA for an additional 24 h before the following experiments.

### Acyl‐RAC Assay

Acyl‐RAC was performed as described.^[^ ^58^ ^]^ After cellular culture manipulations, HUVECs were harvested in 200 µL RIPA lysis buffer with 1% Phenylmethanesulfonyl fluoride (PMSF). Lysates were centrifuged at 12 000 g for 15 min, and the supernatants were collected for further processing. Equal amount of 2× blocking buffer (0.4% S‐methanethiosulfonate, 100 mM HEPES, 1.0 mM EDTA, 2.5% SDS, pH 7.5) was added into proteins and incubated at 40 °C for 15 min with constantly shake in order to irreversibly block cysteine residuals. Proteins were cool down and precipitated with 3‐volume ice cold acetone at ‐20 °C for 45 min followed by centrifuged at 12 000 g for 5 min. Protein pellets were washed with 70% ice cold acetone three times, then dried and suspended in 200 µL binding buffer (100 mM HEPES, 1.0 mM EDTA, 1% SDS, pH 7.5) with PMSF (physical destroying was needed if necessary). 40 µL solution was served as input. Half of remaining proteins were added into 10 mg pre‐washed Activated Thiol‐Sepharose 4B (Sigma) followed by addition of 20 µL 2 M hydroxylamine hydrochloride (+HA, pH = 7.5) or 20 µL 2 M NaCl (‐HA, pH = 7.5). Each sample was rotated at room temperature for at least 2 h. Beads were washed with binding buffer three times, and proteins were eluted by 20 µL 5× loading buffer for Western blot. For mass spectrum (MS) analysis, beads were washed with MS‐grade water five times, and were re‐suspended in 100 µL eluting solution. Proteins were eluted at 60 °C for 45 min with constantly shake and remained for following MS analysis.

### Sample Preparation for MS Analysis

Proteins from Acyl‐RAC were alkylated with 100 mM iodoacetamide (IAA, in 50 mM NaHCO_3_) for 30 min at room temperature. Proteins were digested into peptides by 1 µg µL^−1^ trypsin (Thermo Fisher, 1:50 trypsin to protein ratio) at 37 °C with shake overnight. Proteins digestion was stopped by the addition of trifluoroacetic acid (TFA) at 0.2% final concentration. The peptides were desalinated by C18 column followed by vacuum drying and redissolving with water. Pierce Quantitative Colorimetric Peptide Assay (Thermo Fisher) was performed to measure the concentration of peptide mixtures from tryptic digests. Peptides were diluted to 200 ng µL^−1^ for MS analysis.

### Nano‐LC‐MS/MS Analysis

Peptide samples were analyzed by liquid chromatography tandem mass spectrometry using Easy nLC 1200 system and Q Exactive Plus mass spectrum (Thermo Fisher). Briefly, peptides were separated by reverse chromatography column (C18‐AQ, 1.9 µm, PF360‐75‐10‐N‐5, 20 cm) with mobile phase A as 0.1% formic acid mass spectrometry water and mobile phase B as 0.1% formic acid mass spectrometry water containing 80% acetonitrile. The solvent gradient was set to 0–95 min, 3%→32% (B); 95–105 min, 32%→100% (B); 105–120 min, 100% (B). The flow rate was 200 nL min^−1^. The components separated by chromatographic column were formed electrospray via NSI ion source and detected by mass spectrometer. The spray voltage of NSI was 2.2 kV and the ion transfer column temperature was 275 °C. The acquisition resolution of primary mass spectrometry was 70 000, and the scanning range was 355–1700 m/z. The acquisition resolution of the secondary mass spectrometry was 35 000, the CID collision energy was 27%, the Loop count was 10, the AGC target was 5e4, isolation window was 1.6 m/z, fixed first mass was 120 m/z, and dynamic exclusion was 60.0 s. The data collected by mass spectrometry were analyzed using Protein Discovery 2.2.0.388 software (Thermo Fisher). The protein library was Homo sapiens (SwissProt TaxID = 9606).

### THP‐1 Cells Adhesion Assay

The 24‐well plates were seeded with HUVECs followed by corresponding treatment. At the beginning of adhesion assay, THP‐1 cells were stained by 5 µM calcein AM for 30 min. They were then re‐suspended with fresh ECM and seeded into HUVECs monolayers and co‐cultured for 2 h. Non‐adherent THP‐1 cells were washed with PBS gently and the adherent THP‐1 cells with green fluorescence were captured by EVOS M7000 (Thermo Fisher).

### Immunofluorescence Assay

HUVECs and aortic arch sections were fixed with 4% paraformaldehyde or 95% ethanol for 15 min and blocked with 10% goat serum for 30 min at room temperature. Samples were incubated with primary antibodies at 4 °C overnight which was followed by corresponding secondary antibodies respectively. Fluorescence photographs were captured by laser scanning confocal microscopy using FV300 (Olympus).

### Endothelial Permeability Assay

Fluorescein isothiocyanate (FITC)‐dextran permeation assay. HUVECs were seeded on 6.5‐mm Transwell permeable supports (pore size, 0.4 µm; 3413; Corning). After treatment, 1 mg mL^−1^ FITC‐dextran (40 kDa; FD40S; Sigma) was added to ECM of the transwell upper compartments while the lower compartments were added with 0.5 mL ECM. Along with indicated time, 50 µL solution from the lower compartments was collected and measured by using a fluorometer.

### Trans‐Endothelial Electrical Resistance (TEER) Measurement

HUVECs were grown in 24‐well transwell chambers (Corning, pore area = 0.33 cm^2^). After treatment, TERR was measured by using epithelial volt ohm meter (Millipore, MA, USA), and the value was calculated by (Experimental Resistance – Blank Resistance) × pore area.

### NO Production Measurement

NO content was measured using fluorescent probe 4‐amino‐5‐methylamino‐2′,7′‐difluorofluorescein (DAF‐FM) and Griess reagent as previously described.^[^ ^59^ ^]^

### Endothelial Proliferation Assay

Edu staining was used to analyze the HUVECs proliferation. HUVECs were cultured in ECM incubating 50 µM 5‐ethynyl‐2′‐deoxyuridine (Edu) for 2 h at 37 °C. Detection of Edu signal was achieved with the Cell‐Light Edu Apollo 567 (RiboBio), according to the manufacturer's protocol. The ratio of Edu‐positive cells (red) to all cells was analyzed.

### PKM2 Tetramer Assessment

Before cells being lysed, proteins were cross‐linked by 1% formaldehyde for 10 min and stopped with 125 mM glycine for 5 min. Cells were lysed by RIPA and centrifuged at 12 000 g for 15 min at 4 °C. Supernatant was added with non‐denatured Gel Sample Loading Buffer (P0016; Beyotime) followed by Western blotting.

### Glycolytic Rate Assay

The Seahorse XFe96 Extracellular Analyzer was applied to record glycolytic proton efflux rate (glycopPER), followed by injected with Rotenone/Antimycin (Rot/AA) and 2‐deoxy‐D‐glucose (2‐DG) according to the manufacturer's protocol (103344‐100; Agilent). Data were automatically generated by Wave program and Seahorse XF Glycolytic Rate Assay Report Generator.

### Immunoprecipitation

HUVECs were harvest by IP lysis buffer (P0013; Beyotime) with PMSF. 20 µL lysates were loaded as input, and the remaining parts were incubated with indicated antibodies at 4 °C overnight. Protein A/G agarose beads were used to capture the protein‐antibody immune complex with low‐speed rotation at 4 °C for 4 h. Beads were washed with washing buffer and boiled with loading buffer. All samples were subjected to Western blotting as mentioned below.

### Molecular Dynamics Simulations

Molecular dynamics simulations are performed by Amber 18 software package installed on the Linux platform. The high‐performance server cluster on the platform is composed of two Intel Xeon Platinum 8176 CPU processors, accelerated by two NVIDIA Tesla V100 SXM2 Gpus. Proteins and palmitic acid were performed in FF14SB force fields and FBP was performed in GAFF2 force fields. the whole system was solvated in a periodic water box using the TIP3P water model, and was minimizing the palmitoylated PKM2 by using the steepest descent algorithm. After the minimization, the system was heated from 10 K to 310 K with a 200‐ps NVT simulation and equilibrated with a 200‐ps NPT simulation. Finally, a 10‐ns production simulation was conducted in the NPT ensemble. The trajectory of the simulation was analyzed using various tools provided by the CPPTRAJ package of Amber 18, including the protein root mean square deviation (RMSD) and root mean square fluctuation (RMSF).

### Molecular Docking

The crystal structure (PDB: ET5A) of PKM2 was downloaded from the protein data bank. The Glide‐grid of total 10 cysteins was generated using the Receptor Grid Generation module. Palmitoyl‐CoA was subjected to the LigPrep module to apply forth field (OPLS‐2005z). Molecular docking was performed using Glide Covalent Docking Module.

### Western Blotting

HUVECs were lysed on ice with RIPA buffer (P0013B; Beyotime) with PMSF and centrifuged at 12 000 g for 15 min at 4 °C. Protein concentration was determined by BCA protein assay kit (23 227; Thermo Fisher) and separated by SDS‐PAGE and transferred to PVDF membrane (ISEQ85R; Millipore). The membrane was blocked by 5% non‐fat milk and incubated with indicated primary antibodies at 4 °C overnight, besides corresponding secondary antibodies at room temperature for 2 h, which was followed by chemiluminescent detection (180‐506; Tanon). Image blots were captured by chemical imaging system (Tanon) and analyzed by Image J software (v1.53, National Institutes of Health, USA).

### Quantitative Real‐Time PCR

The total RNA was extracted by using Trizol (Invitrogen) lysis reagent. 1–2 mg RNA was reverse‐transcribed into cDNA according to the manufacture of reverse transcription kit (Thermo Fisher). With SYBR Green Quantitative PCR kit and specific primers listed in Table S4 (Supporting Information), cDNA was amplified by Real‐time quantitative PCR system using lightCycler480II (Roche). Primers for target genes were synthesized by Sangon Biotech Co., Ltd (Shanghai, China). Gene expression was normalized to glyceraldehyde 3‐phosphate dehydrogenase (GAPDH).

### Statistical Analysis

For cohort study, Pearson correlation analysis and univariate linear regression analysis were used to investigate the correlation between serum palmitic acid concentration and blood glucose as well as lipid levels in patients with CHD. Additionally, restricted cubic splines and Cox survival analysis were utilized to further explore the relationship between palmitic acid and the risk of mortality in CHD patients. For cell and animal studies, all data were presented as mean ±SD. When data passed normality and equal variance test, unpaired two‐tailed Student's t‐test was used for comparisons between two groups while one‐way ANOVA with Tukey's multiple comparisons test as the post hoc test was used to compare multiple groups. *P*‐values < 0.05 were considered statistically significant. Statistical analyses were conducted with GraphPad Prism 8.0 software.

## Conflict of Interest

The authors declare no conflict of interest.

## Author Contributions

Yu He: Designed the study, performed animal and cell experiments, analyzed the data, and drafted the manuscript. Senlin Li: Analyzed the clinical cohort study and drafted the clinical study part of the manuscript. Lujing Jiang: Participated in experimental design and provided technical support. Kejue Wu: Participated in molecular dynamics simulations and molecular docking analysis. Shanshan Chen: Participated in experimental design and revised the manuscript. Linjie Su: Participated in the measurement of endothelial permeability and revised the manuscript. Cui Liu: Participated in the experiments to eliminate the involvement of BSA and revised the manuscript. Peiqing Liu: Designed the study, reviewed, and revised the manuscript. Wenwei Luo: Contributed to clinical data analysis and revised the manuscript. Shilong Zhong: Responsible for the clinical cohort study. Contributed to patient recruitment and clinical data collection, reviewed and revised the manuscript. Zhuoming Li: The principal investigator of this study. Contributed to the overall study design, data analysis, and manuscript written, and revision.

## Supporting information



Supporting Information

## Data Availability

The data that support the findings of this study are available from the corresponding author upon reasonable request.
