# Supplementary material for: Palmitic Acid Accelerates Endothelial Cell Injury and Cardiovascular Dysfunction via Palmitoylation of PKM2
Source: Adv Sci (Weinh). 2024 Dec 12;12(5):2412895. doi: 10.1002/advs.202412895 (PMC11791964; doi:10.1002/advs.202412895)

**Supplementary method**

**Cohort study.** Our study involved the participation of 1040 individuals who underwent percutaneous coronary intervention (PCI) at Guangdong Provincial People's Hospital, with follow-up tracking. All participants were followed up prospectively for the study endpoints based on inpatient and outpatient hospital visits and telephone contacts with the patients or their families. At each follow-up assessment (every 6 months), the participants were questioned about new adverse cardiovascular events. Patients were sequentially enrolled from Guangdong Provincial People’s Hospital between January 2010 and December 2013 and followed up for the primary endpoint (all-cause death) and secondary endpoint (MACE) from June 2010 through April 2017 for a median of 3.5 years, with 63 deaths and 183 MACEs.

Enrollment criteria targeted patients recommended for diagnostic coronary angiography or PCI due to CHD, specifically those with at least one major coronary artery showing ≥50% luminal obstruction. CHD patients were further categorized into stable CHD and acute coronary syndrome (ACS), the latter diagnosed per ACC/AHA guidelines encompassing unstable angina, ST-elevation myocardial infarction (MI), and Non-ST elevation MI. Exclusion criteria eliminated individuals under 18 or over 80 years old, those with significantly high serum creatinine or transaminase levels, undergoing pregnancy or breastfeeding, in advanced cancer stages or with a history of haemodialysis, with recent thyroid issues or medication, and anyone lost to follow-up (Figure S10).

Comprehensive metabolomic profiling was performed across the cohorts, employing UPLC (Shim-pack UFLC SHIMADZU CBM30A) and MS (Applied Biosystems 4500 QTRAP), facilitated by Wuhan Metware. Variations in the metabolomic platform and methodology, such as updates to the MS system, modifications in ESI source operation parameters, alterations in flow rate, and injection volume differences, resulted in discrepancies in metabolite detection capabilities between the cohorts. Consequently, cohort yielded detections of 600 metabolites, and the metabolomic data was scaled using Pareto scaling.

Pearson correlation analysis and univariate linear regression analysis were used to investigate the correlation between serum palmitic acid concentration and blood glucose as well as lipid levels in patients with CHD. Additionally, restricted cubic splines and Cox survival analysis were utilized to further explore the relationship between palmitic acid and the risk of mortality in CHD patients.

Mediation analysis was used to identify mediation factors using the mediation package. The total effect was estimated by a logistic regression model. The effect of palmitic acid on the mediator was assessed by a linear regression model. The direct and indirect effects were then evaluated using a predictive model. The mediation analysis was performed using the mediate function from the mediation package. Bootstrapping (boot=T) with 1000 simulations (sims=1000) was used to estimate confidence intervals for the mediation effects.

| **Table S1 Baseline characteristics in 1040 CHD patients** | |
| --- | --- |
| **Characteristics** | **Overall (n = 1040)** |
| Age | 63.034 ±10.045 |
| Sex (male) | 828 (79.62) |
| BMI, kg/m^2^ | 24.262±4.781 |
| SBP, mm Hg | 130.659±18.887 |
| DBP, mm Hg | 76.185 ±11.028 |
| Current smoking | 294 (28.54) |
| Family of CVD | 29 (2.79) |
| Comorbidities |  |
| Arrhythmia | 92 (8.86) |
| DM | 286 (27.55) |
| HyperT | 412 (39.65) |
| Dyslipidemia | 729 (72.54) |
| Biomedical measurements | |
| ALT, U/L | 27.412 ±13.181 |
| AST, U/L | 26.639 ±10.623 |
| eGFR, mL/min/1.73 m2 | 94.318 ±73.690 |
| GLUC, mmol/L | 6.741 ±2.729 |
| CHOL, mmol/L | 4.278 ±1.125 |
| LDLC, mmol/L | 2.579 ±0.926 |
| HDLC, mmol/L | 0.965 ±0.257 |
| TRIG, mmol/L | 1.616 ±1.136 |
| CKMB, U/L | 7.476 ±5.920 |
| proBNP, pg/mL | 774.514 ±1597.349 |
| WBC,10^9/L | 7.499 ±2.104 |
| hs-CRP, mg/L | 8.334 ±17.178 |
| FIB, g/L | 3.407 ±3.238 |
| CRP, mg/L | 10.952 ±25.263 |
| Medications | |
| BB | 929 (89.50) |
| ACEI | 660 (63.58) |
| CCB | 295 (28.42) |
| PPI | 506 (48.75) |
| SYNTAX score | 16.433 (10.738) |
| LVEF, % | 60.102 ±11.539 |
| LVMI, g/m^2^ | 122.129 ±36.046 |
| SII | 601.279 (458.844) |
| PLR | 126.558 (59.008) |
| NLR | 2.735 (1.724) |
| LMR | 4.469 (14.107) |
| Data are number (%) or mean ± SD when appropriate | |
| *SD* standard deviation, *BMI* body mass index, *SBP* systolic blood pressure, *CVD* cardiovascular disease,  *DM* diabetes, *HyperT* hypertension, *ALT* alanine aminotransferase, *AST* aspartate aminotransferase,  *eGFR* estimated glomerular filtration rate, *GLUC* glucose, *CHOL* cholesterol, *LDLC* low-density lipoprotein  cholesterol, *HDLC* high-density lipoprotein cholesterol, *TRIG* triglyceride, *CKMB* creatine kinase MB,  *proBNP* N-terminal pro brain natriuretic peptide, *BB* β-blockers, *ACEI* angiotensin converting enzyme inhibitors,  *CCB* calcium channel blockers, *PPI* proton pump inhibitors, *SYNTAX* *score* Synergy between PCI with TAXUS and  Cardiac Surgery score, *LVEF* left ventricular ejection fraction, *LVMI* left ventricular mass index*, WBC* whole plasma  cell count, *LMR* lymphocyte-monocyte ratio, *NLR* neutrophil-lymphocyte ratio, *PLR* platelet-lymphocyte ratio, *FIB* fibrinogen | |

| **Table S2**. The mediation analysis between PA and Death and MACE | | | | | | | | | | |
| --- | --- | --- | --- | --- | --- | --- | --- | --- | --- | --- |
|  | Direct Effect | | |  | Indirect Effect | |  | Total Effect | | |
|  | β(95%CI) | p-Value | |  | β(95%CI) | p-Value |  | β(95%CI) | p-Value | |
| Death | -0.003  (-0.057- 0.051) | | 0.854 |  | 0.006  (0.001- 0.017) | 0.014 |  | 0.003  (-0.051- 0.055) | | 0.964 |
|  |  | |  |  |  |  |  |  | |  |
| MACE | 0.053  (-0.016- 0.121) | | 0.136 |  | 0.004  (-0.006-0.018) | 0.456 |  | 0.058  (-0.015 -0.124) | | 0.106 |

**Table S3**

| STROBE Statement—Checklist of items that should be included in reports of ***cohort studies*** | | | |
| --- | --- | --- | --- |
|  | **Item No** | **Recommendation** | **Page No** |
| **Title and abstract** | 1 | (*a*) Indicate the study’s design with a commonly used term in the title or the abstract | 2 |
|  |  | (*b*) Provide in the abstract an informative and balanced summary of what was done and what was found | 2 |
| **Introduction** | | | |
| Background/rationale | 2 | Explain the scientific background and rationale for the investigation being reported | 4-5 |
| Objectives | 3 | State specific objectives, including any prespecified hypotheses | 4-5 |
| **Methods** | | | |
| Study design | 4 | Present key elements of study design early in the paper | Supplementary method |
| Setting | 5 | Describe the setting, locations, and relevant dates, including periods of recruitment, exposure, follow-up, and data collection | Supplementary method |
| Participants | 6 | (*a*) Give the eligibility criteria, and the sources and methods of selection of participants. Describe methods of follow-up | Supplementary method |
|  |  | (*b*) For matched studies, give matching criteria and number of exposed and unexposed | N/A |
| Variables | 7 | Clearly define all outcomes, exposures, predictors, potential confounders, and effect modifiers. Give diagnostic criteria, if applicable | 5,  Table S1 |
| Data sources/ measurement | 8* | For each variable of interest, give sources of data and details of methods of assessment (measurement). Describe comparability of assessment methods if there is more than one group | Supplementary method |
| Bias | 9 | Describe any efforts to address potential sources of bias | Supplementary method |
| Study size | 10 | Explain how the study size was arrived at | Supplementary method |
| Quantitative variables | 11 | Explain how quantitative variables were handled in the analyses. If applicable, describe which groupings were chosen and why | Supplementary method |
| Statistical methods | 12 | (*a*) Describe all statistical methods, including those used to control for confounding | Supplementary method |
|  |  | (*b*) Describe any methods used to examine subgroups and interactions | N/A |
|  |  | (*c*) Explain how missing data were addressed | Supplementary method |
|  |  | (*d*) If applicable, explain how loss to follow-up was addressed | Supplementary method |
|  |  | (*e*) Describe any sensitivity analyses | N/A |
| **Results** | | |  |
| Participants | 13* | (a) Report numbers of individuals at each stage of study—eg numbers potentially eligible, examined for eligibility, confirmed eligible, included in the study, completing follow-up, and analysed | Supplementary method |
|  |  | (b) Give reasons for non-participation at each stage | Supplementary method |
|  |  | (c) Consider use of a flow diagram | Figure S10 |
| Descriptive data | 14* | (a) Give characteristics of study participants (eg demographic, clinical, social) and information on exposures and potential confounders | Supplementary method |
|  |  | (b) Indicate number of participants with missing data for each variable of interest | Figure S10 |
|  |  | (c) Summarise follow-up time (eg, average and total amount) | Supplementary method |
| Outcome data | 15* | Report numbers of outcome events or summary measures over time | Supplementary method and Figure S10 |

**Table S4. List of primers for qPCR analysis.**

| Genes | Primer (Forward) | Primer (Reverse) |
| --- | --- | --- |
| *GAPDH* | GGATTTGGTCGTATTGGG | GGAAGATGGTGATGGGATT |
| *Pkm2* | ACTGGCATCATCTGTACCATTG | AGCCACATTCATTCCAGACTTA |
| *Zdhhc2* | TGGCTCTGGGACTCTCTCAATGG | TGATGGCTGCTCCTTTCCAACTTG |
| *Zdhhc4* | TGGACTTGGGGCTCTGCTGAC | CCTGGGTGGCTACCTGTTGTTTG |
| *Zdhhc5* | TTCAGACAGCCCTGATTTTGAG | TGACAGGAAGGGAGAGGTATAG |
| *Zdhhc8* | GTGACAACTGTGTAGAGGACTT | GGAACAGGAAGAAGTAGCGATA |
| *Zdhhc9* | GTTACACATGCAAGATCTTCCG | GTAGAAGTAGCGGTAGTTCCTC |
| *Zdhhc12* | TTCCTCCAGCCATCCCTCTTCG | TCTCCCACACAGTTCTCCATCCAG |
| *Zdhhc13* | GGTCACTCCACTGCCATGTATGC | ACCTATGCACCGTCCAGTCCAC |
| *Zdhhc16* | CAATTGTGTGGGCCACTATAAC | GTTCTTGTCGAGCTGTTTCATT |
| *Zdhhc17* | GCAGGGAATACCACAGTCATTAGCC | TGCTTGCCTTGCCTCTTGTAAGTG |
| *Zdhhc20* | ATGTTCTTCATCAGCGTCCTCTCAC | GGTGCGCGGAATGATTCTATTGTTG |
| *Zdhhc21* | CACTCGTTGGAAGATCCTGTGGTTC | AGACATGATTGGCAAAGTGGTAGGG |
| *IL-6* | GCCTTCGGTCCAGTTGCCTTC | GTTCTGAAGAGGTGAGTGGCTGTC |
| *IL-8* | CTCTCTTGGCAGCCTTCCTGATTTC | GGGGTGGAAAGGTTTGGAGTATGTC |
| *IL-1β* | GGACAGGATATGGAGCAACAAGTGG | TCATCTTTCAACACGCAGGACAGG |
| *ICAM-1* | GTCACCTATGGCAACGACTCCTTC | AGTGTCTCCTGGCTCTGGTTCC |

**Table S5. The sequence of siRNA used for RNA interference**

| **Target gene** | **Target sequence** |
| --- | --- |
| Negative control siRNA | UUCUCCGAACGUGUCACGUTT |
| *Zdhhc5* siRNA | GTGCCCATCTACAATGCAA |
| *Zdhhc9* siRNA | GGAACACCTTTTGCTGTGA |
| *Zdhhc13* siRNA | GAAGGTTCTTCGCTCAGTA |


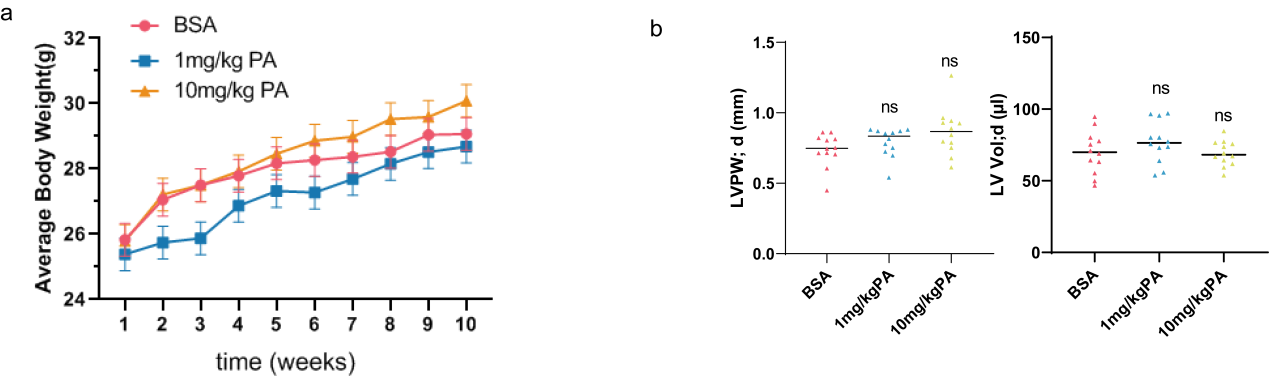


**Figure S1 PA induced endothelial inflammation and atherosclerosis in ApoE^-/-^ mice.** ApoE^-/-^ mice aged 6~8 weeks were divided into three groups and intraperitoneally administrated with BSA or 1 mg/kg PA or 10mg/kg PA individually for 10 weeks. (a) Body weight change over 10 weeks (n=12). (b) Statistic results of left ventricular posterior wall (LVPW) and left ventricular end-diastolic volume (LV vol; d) (n=12). ns means no significant. Statistical significance was calculated using one-way ANOVA. Data are shown as mean ±SD. ns means no significance.


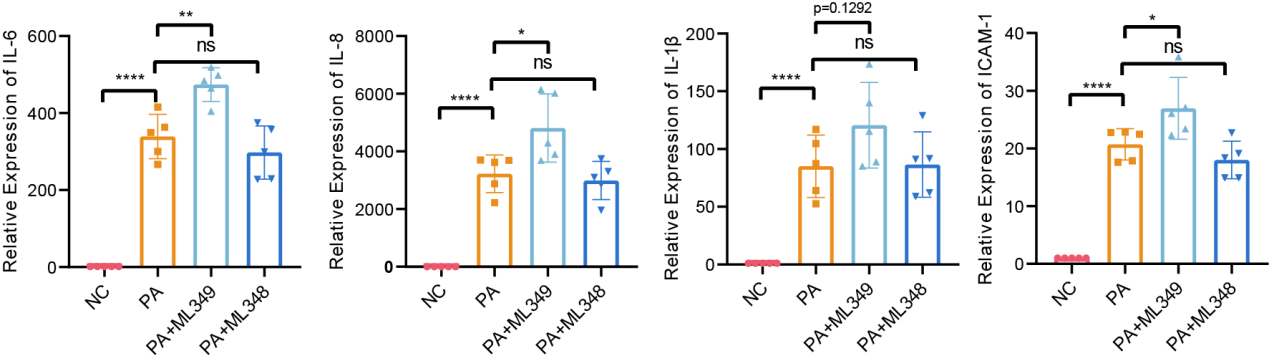


**Figure S2** Relative mRNA level of *IL-6*, *IL-8*, *IL-1β* and *ICAM-1* were measured (n=5). Statistical significance was calculated using one-way ANOVA. Data are shown as mean ±SD. **P* < 0.05; ***P* < 0.01; *****P* < 0.0001; ns means no significance.


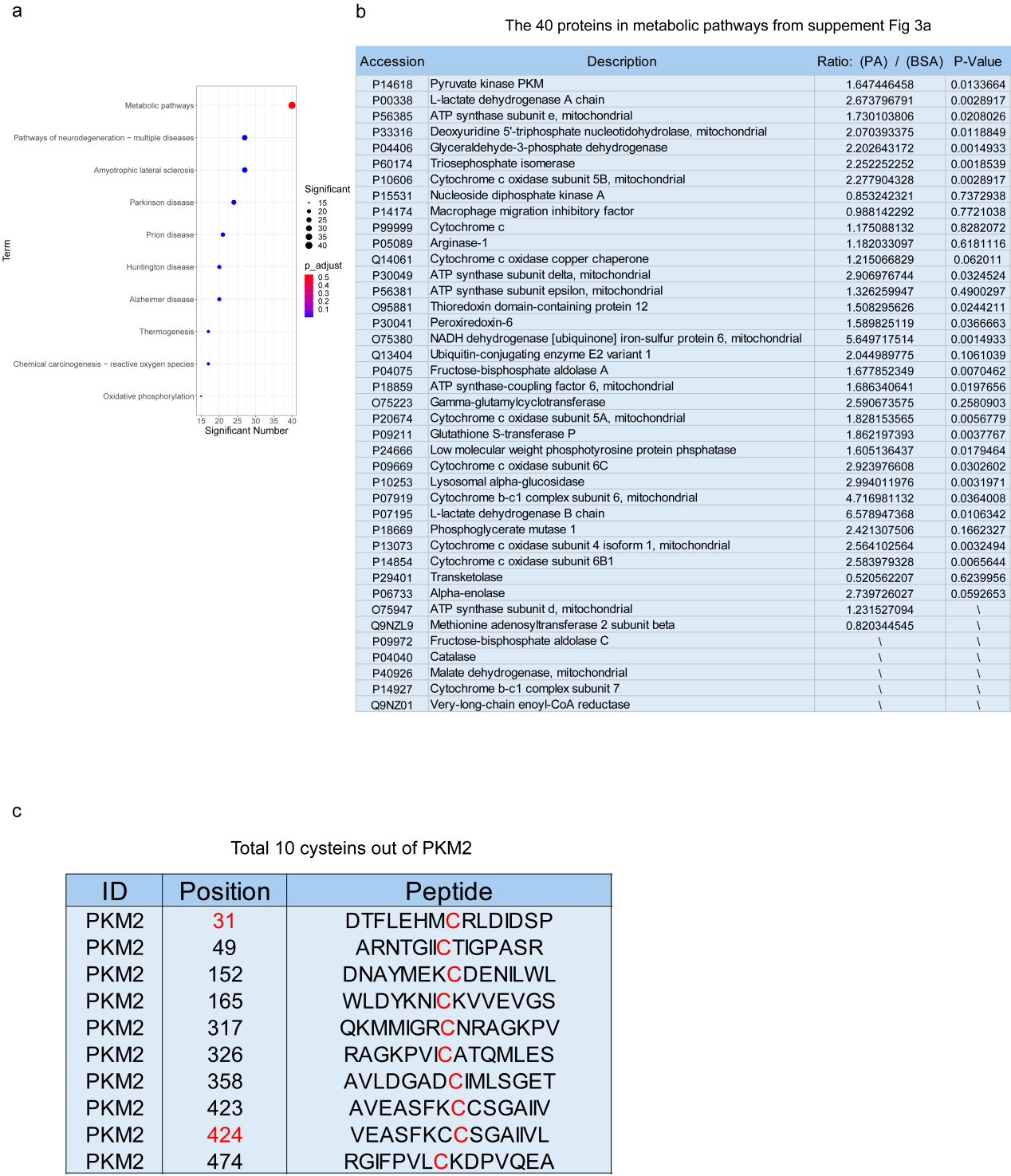


**Figure S3 Palmitoylation proteomic analysis and PKM2 cysteins sites.** (a) KEGG analysis of palmitoylation proteomic results. Sort by number of moleculars. (b) The 40 proteins with their abundance ratio and p-value classified as metabolic pathway. (c) Total 10 cysteins in PKM2, which were analyzed by CSS-Palm 4.0 and molecular docking. Cys31 and Cys424 were the only two with enough space for interacting with palmitoyl-CoA.


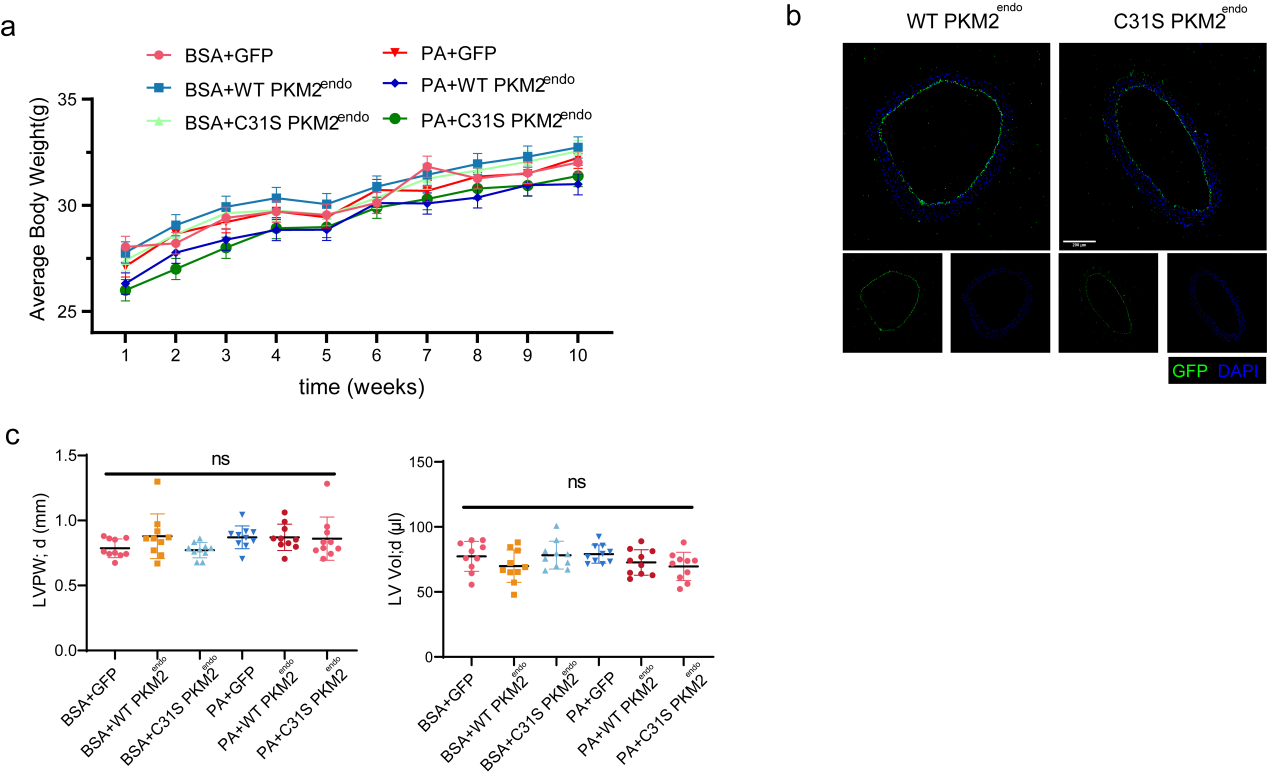


**Figure S4 Vascular endothelial C31S PKM2 mitigated PA-induced cardiovascular dysfunction.** ApoE^-/-^ mice aged 6~8 weeks were divided into six groups and intraperitoneally administrated with BSA or 10 mg/kg PA individually for 10 weeks as well as tail intravenously injected with endothelial specifically expressed WT or C31S PKM2 once every five weeks. (a) Body weight change over 10 weeks (n=10). (b) Respective images of aortic arch developed with blue (DAPI) and green (EC-enhanced AAV vector containing GFP). Scale bars, 200 μm. (c) Statistic results of left ventricular posterior wall (LVPW) and left ventricular end-diastolic volume (LV vol; d) (n=10). Statistical significance was calculated using one-way ANOVA. Data are shown as mean ±SD. ns means no significance.


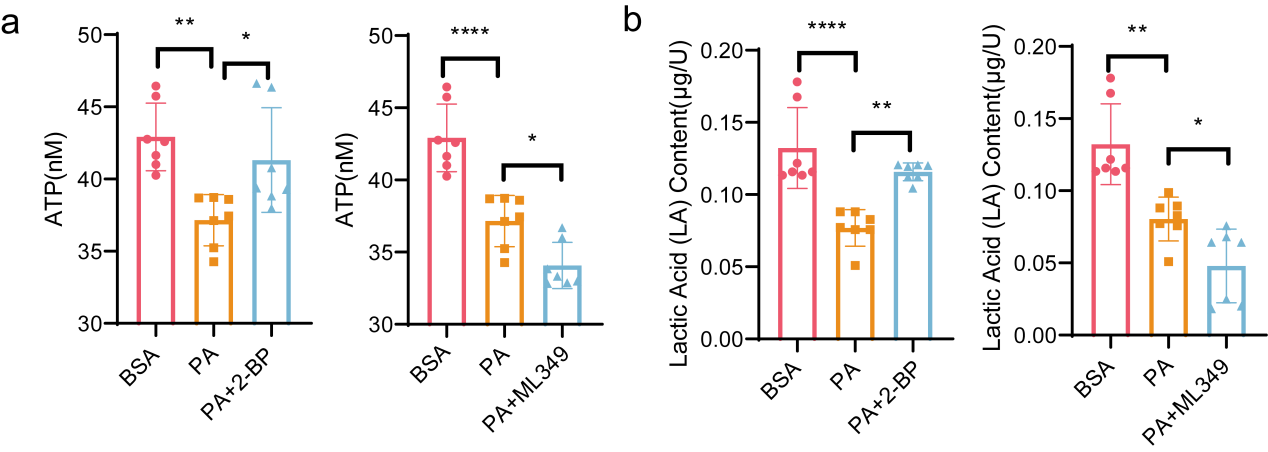


**Figure S5 Endothelial palmitoylation regulated PA-induced inflammation via inhibiting glycolysis.** (a-b) HUVECs were treated with PA and either 2-BP or ML349. Statistic of ATP and lactic acid level in HUVECs (n=7). Statistical significance was calculated using one-way ANOVA. Data are shown as mean ±SD. **P* < 0.05; ***P* < 0.01; *****P* < 0.0001.


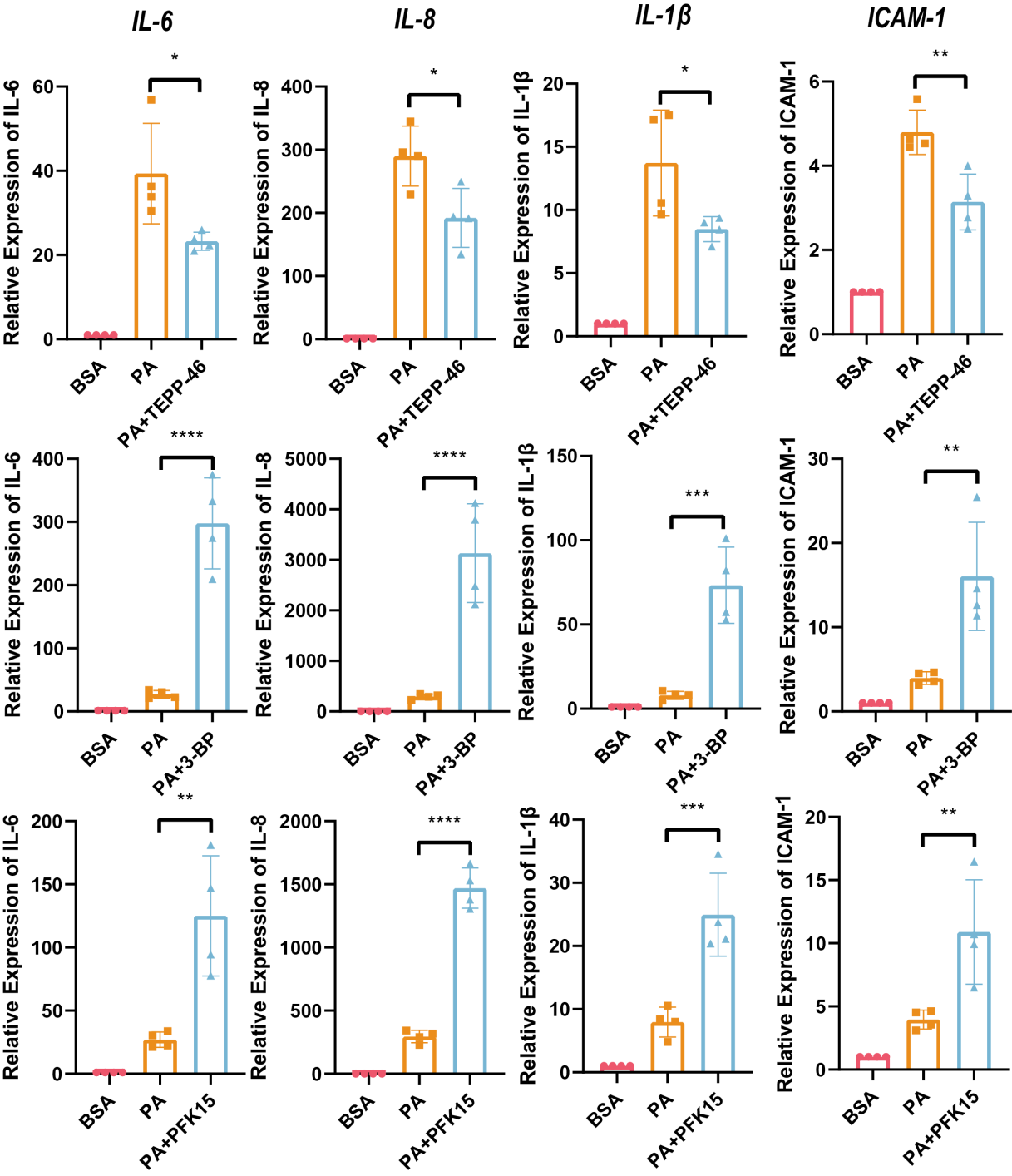


**Figure S6 Glycolytic inhibitors or activators mediated endothelial inflammation.** Relative mRNA level of *IL-6, IL-8, IL-1β* and *ICAM-1* were measured. HUVECs were treated with PA and 20 μM TEPP-46 or 10 μM 3-BP or 5 μM PFK15 for 24 h (n=4). Statistical significance was calculated using one-way ANOVA. Data are shown as mean ±SD. **P* < 0.05; ***P* < 0.01; ****P* < 0.001; *****P* < 0.0001.

**
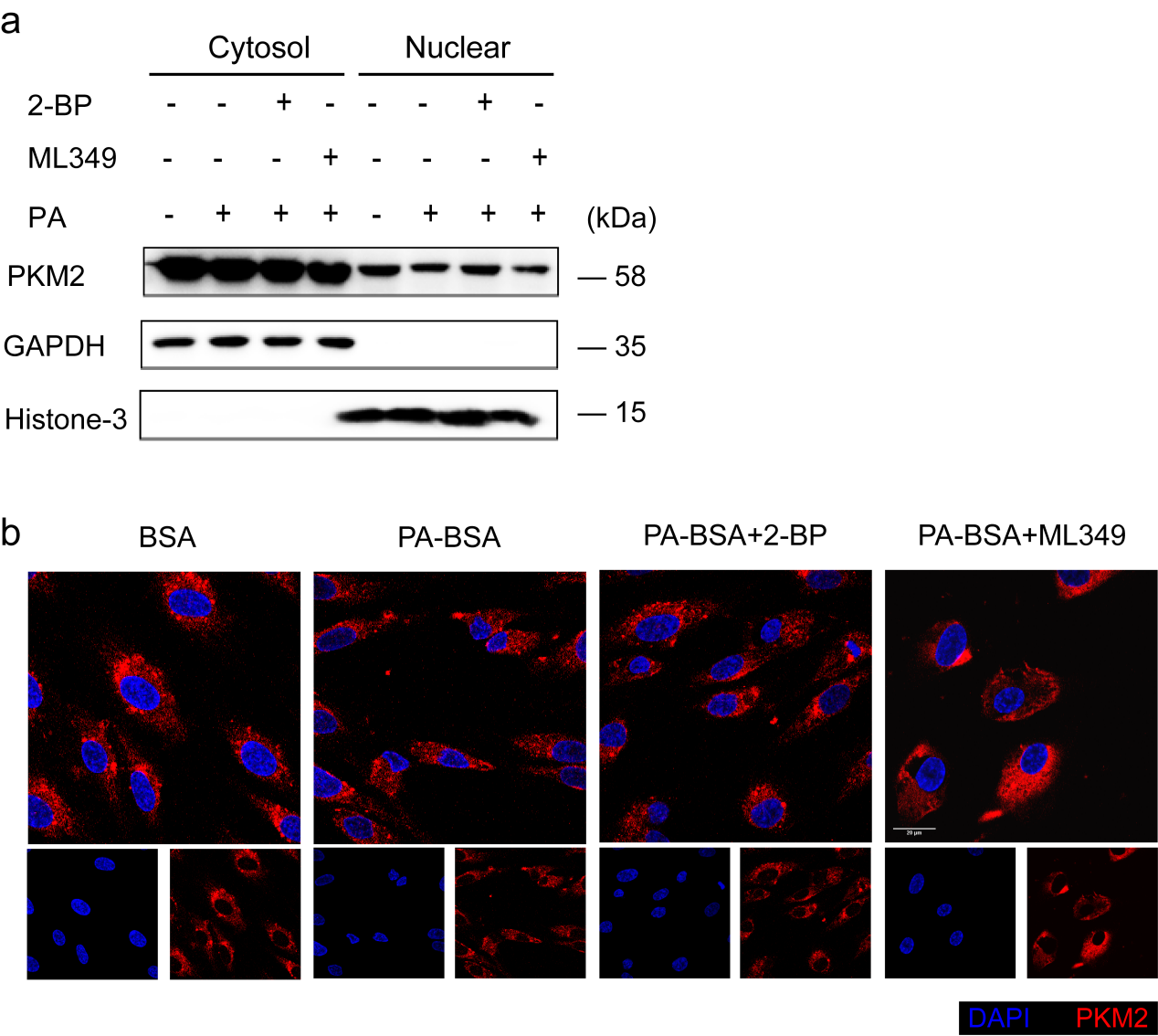
**

**Figure S7 PKM2 nuclear translocation.** (a) Representative images of immunoblots to assess PKM2 nuclear translocation. HUVECs proteins were divided into cytosol and nuclear followed by Western blot analysis (n=3). (b) Representative images of immunofluorescence to study PKM2 (red) nuclear (blue) translocation (n=5). Scale bars, 20 μm. Statistical significance was calculated using one-way ANOVA.


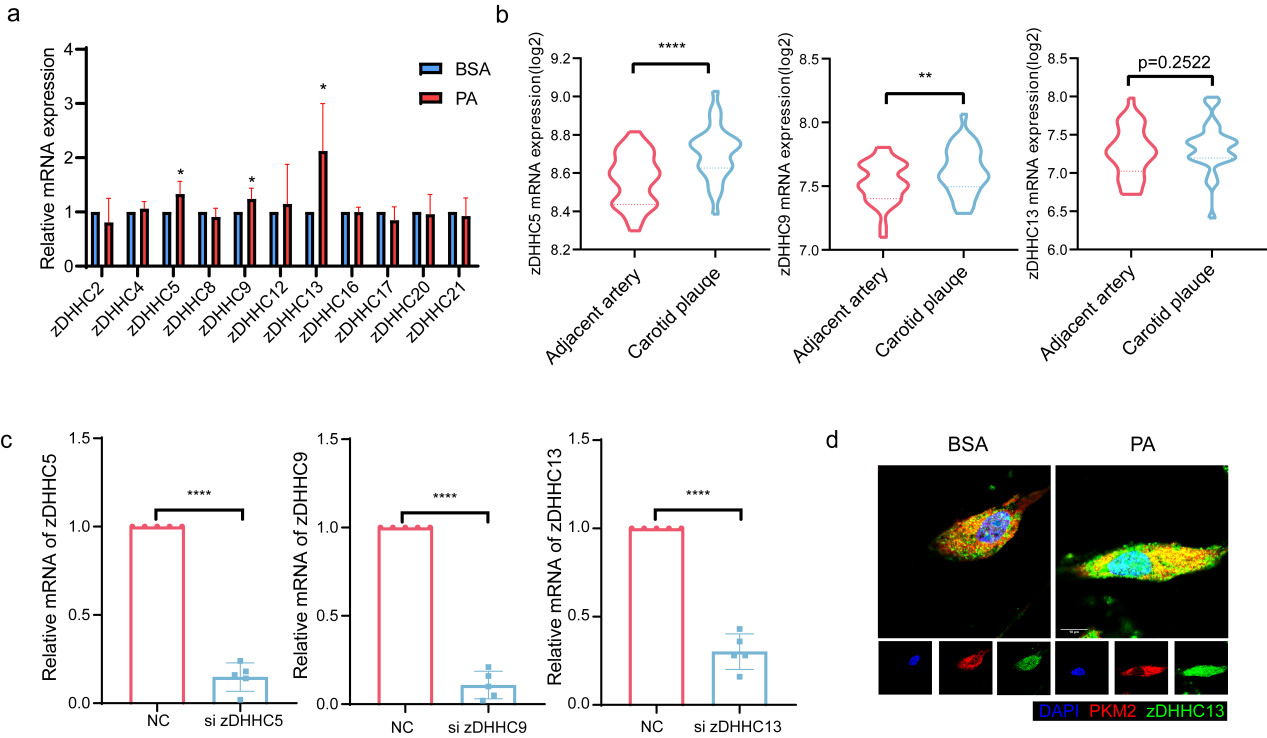


**Figure S8 zDHHC13 palmitoylated PKM2 leading to endothelial inflammation via inhibition of glycolysis.** (a) The relative mRNA level of zDHHCs in PA treated HUVECs (n=4). (b) The relative expression of zDHHC5, zDHHC9 and zDHHC13 in the GEO set, GSE432092. (c) Relative mRNA expression of zDHHC5, zDHHC9 and zDHHC13 after silencing in HUVECs (n=5). (d) Representative images of immunofluorescence of zDHHC13 (green), PKM2 (red) and DAPI (blue) (n=5). Scale bars, 10 μm. Statistical significance was calculated using two-sided Student’s t-tests. Data are shown as mean ±SD. **P* < 0.05; ***P* < 0.01; *****P* < 0.0001.


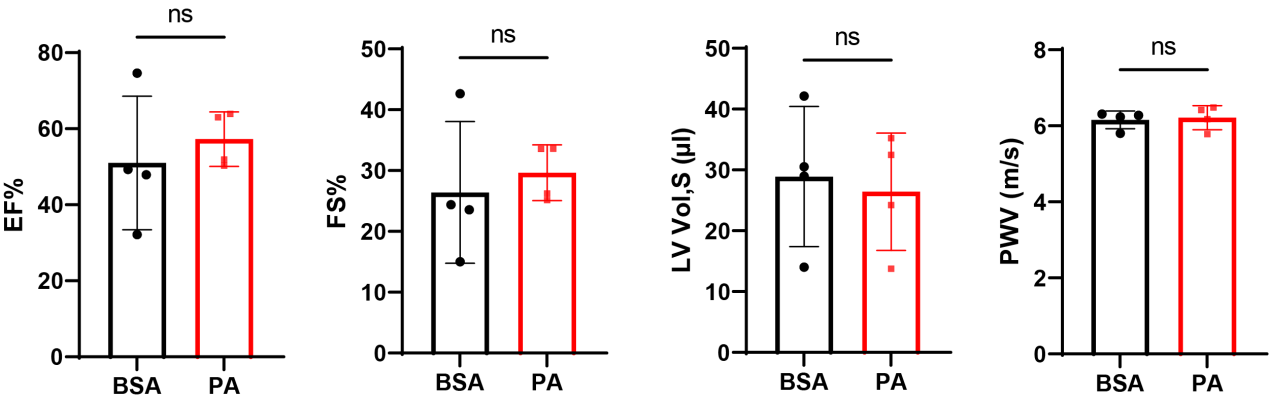


**Figure S9** C57BL/6 mice were intraperitoneally injected with 100 mg/kg PA-BSA for 4 weeks. Statistic results of echocardiographic measurements of fractional shortening (FS), ejection fraction (EF), left ventricular end-systolic volume (LV vol; s) and pulse wave velocity (PWV) (n=5). Statistical significance was calculated using two-sided Student’ s t-tests; Data are shown as mean ±SD. ns means no significance.


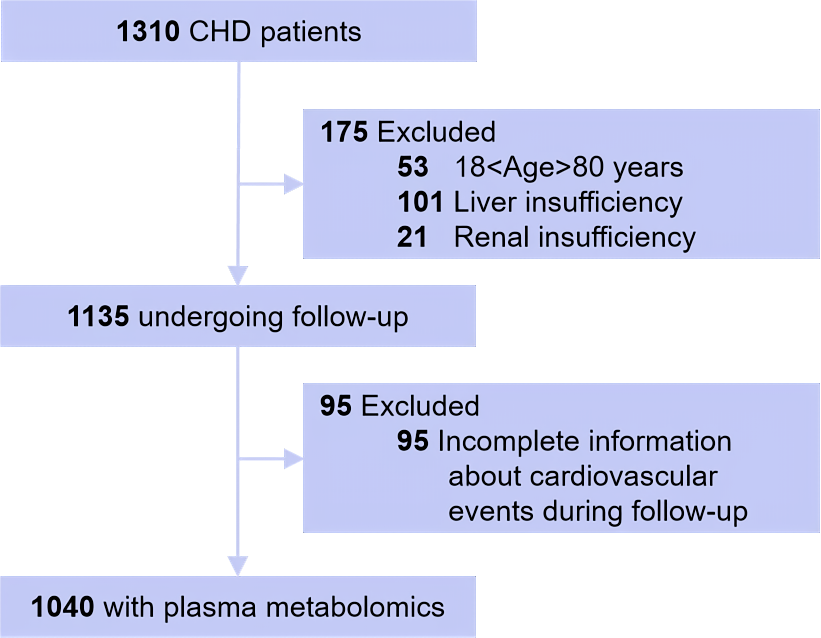


**Figure S10** Workflow chart of data generation and analysis.


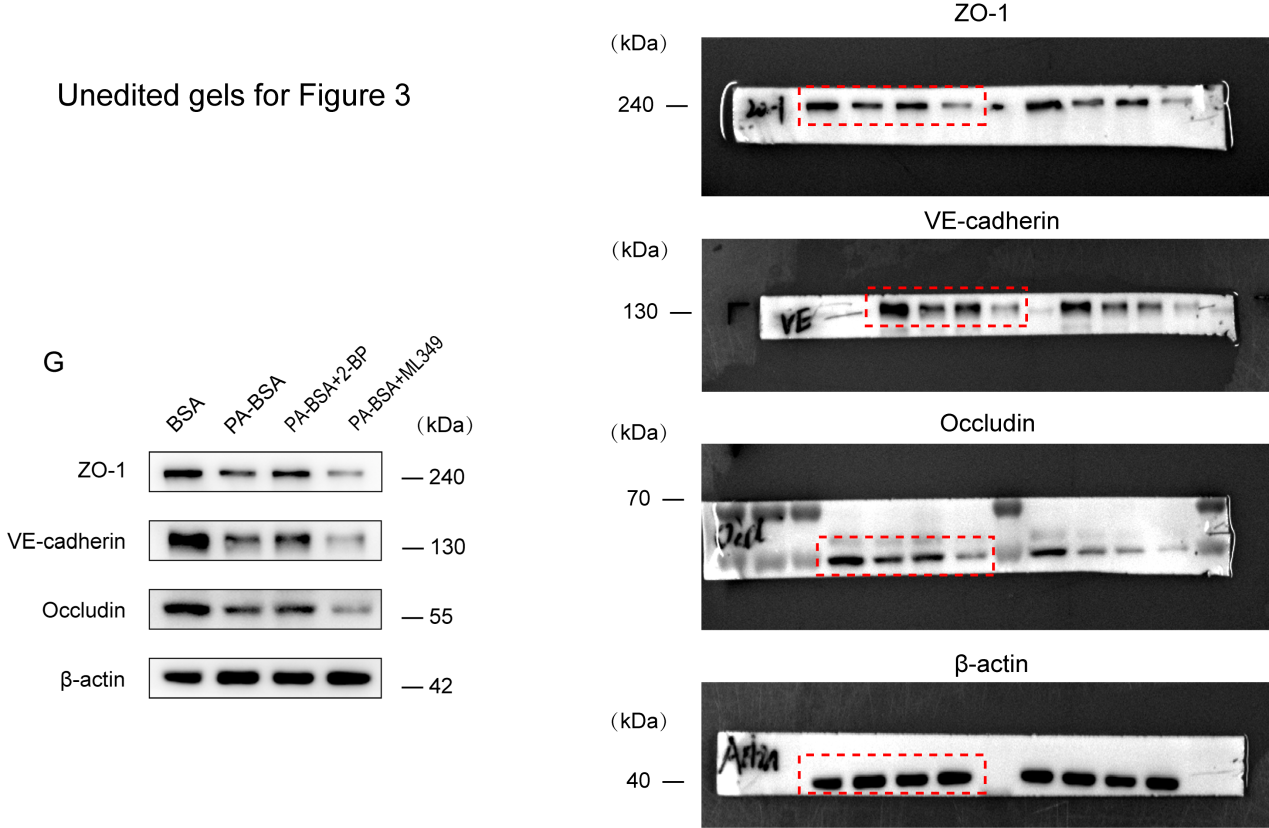


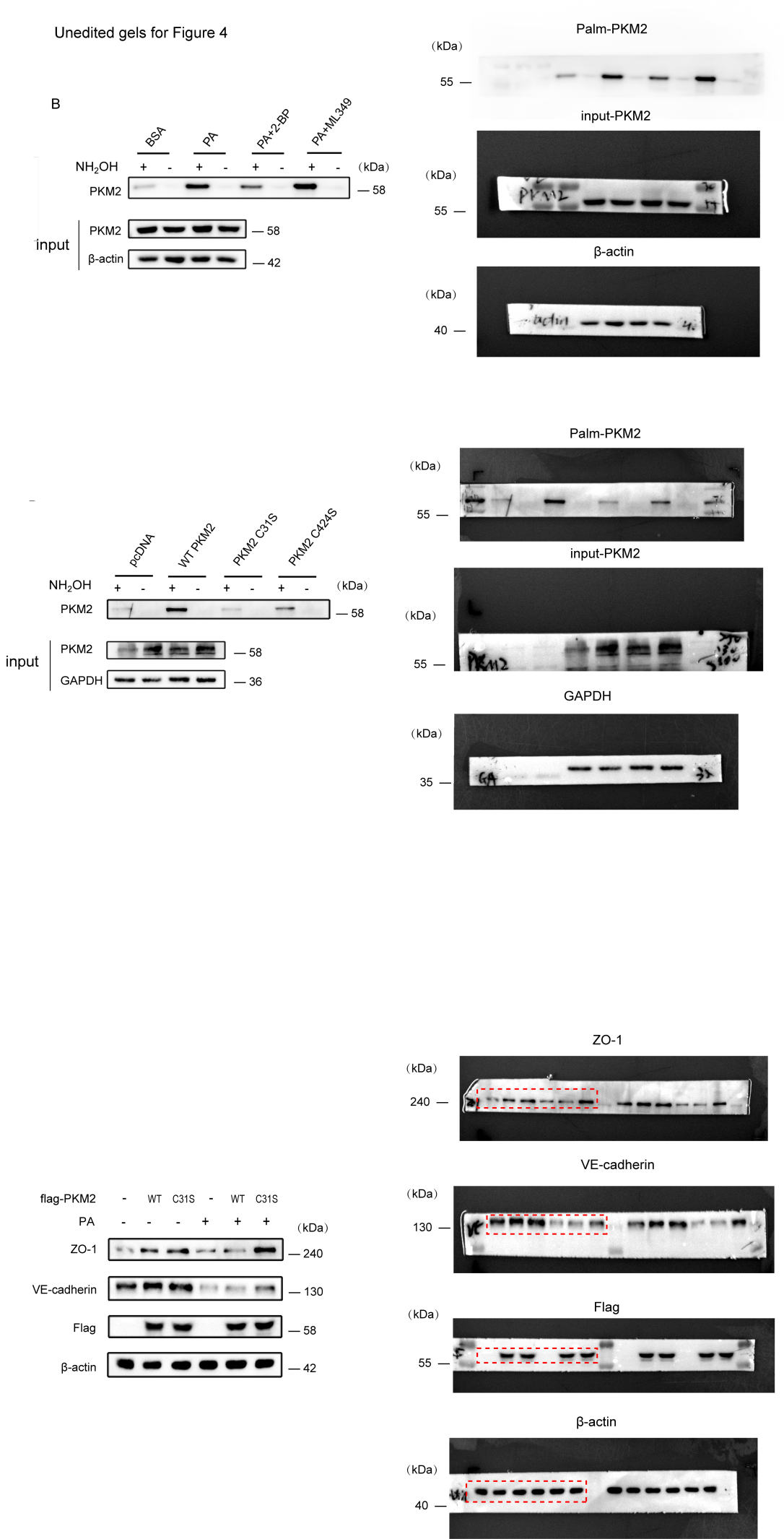


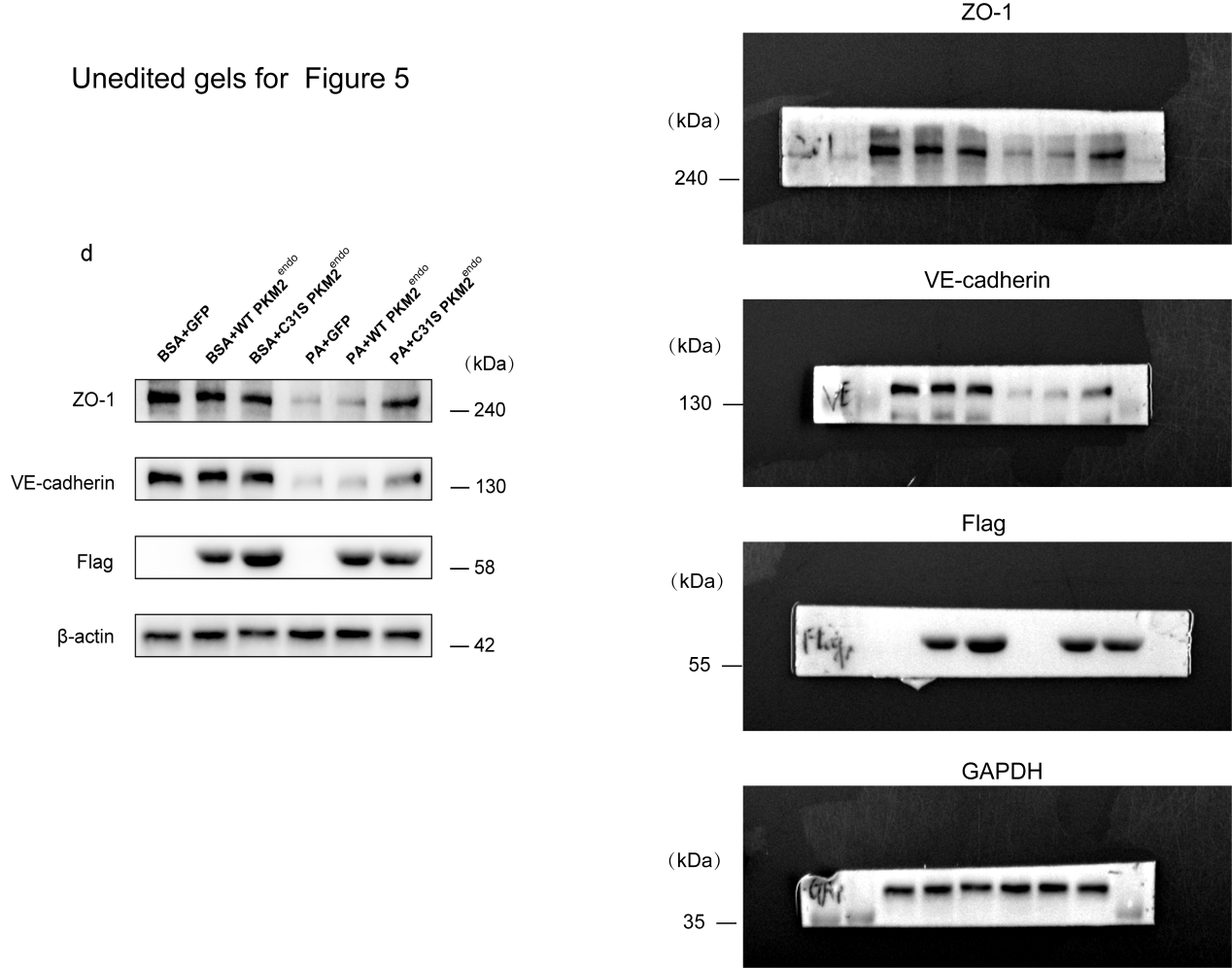


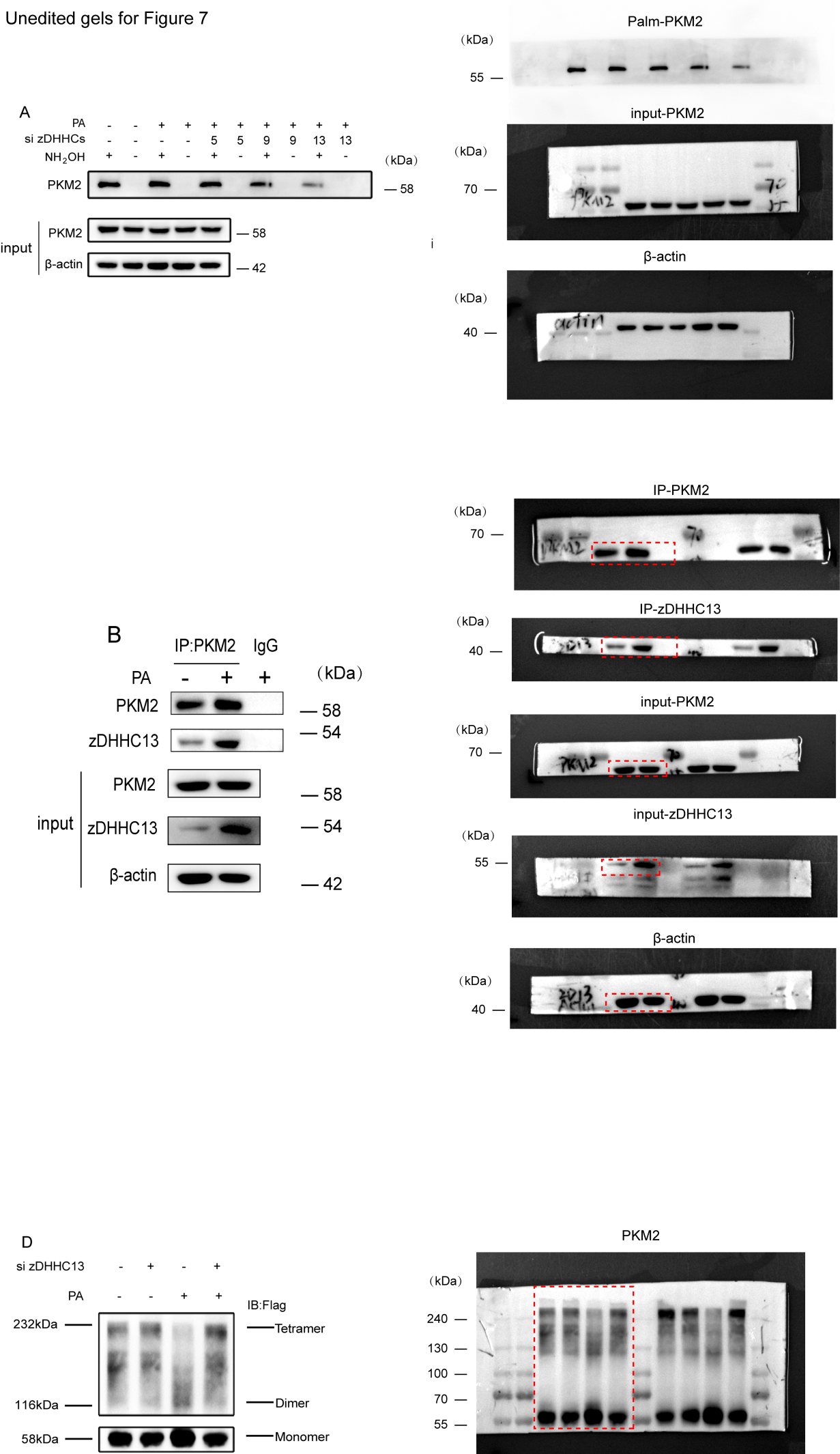


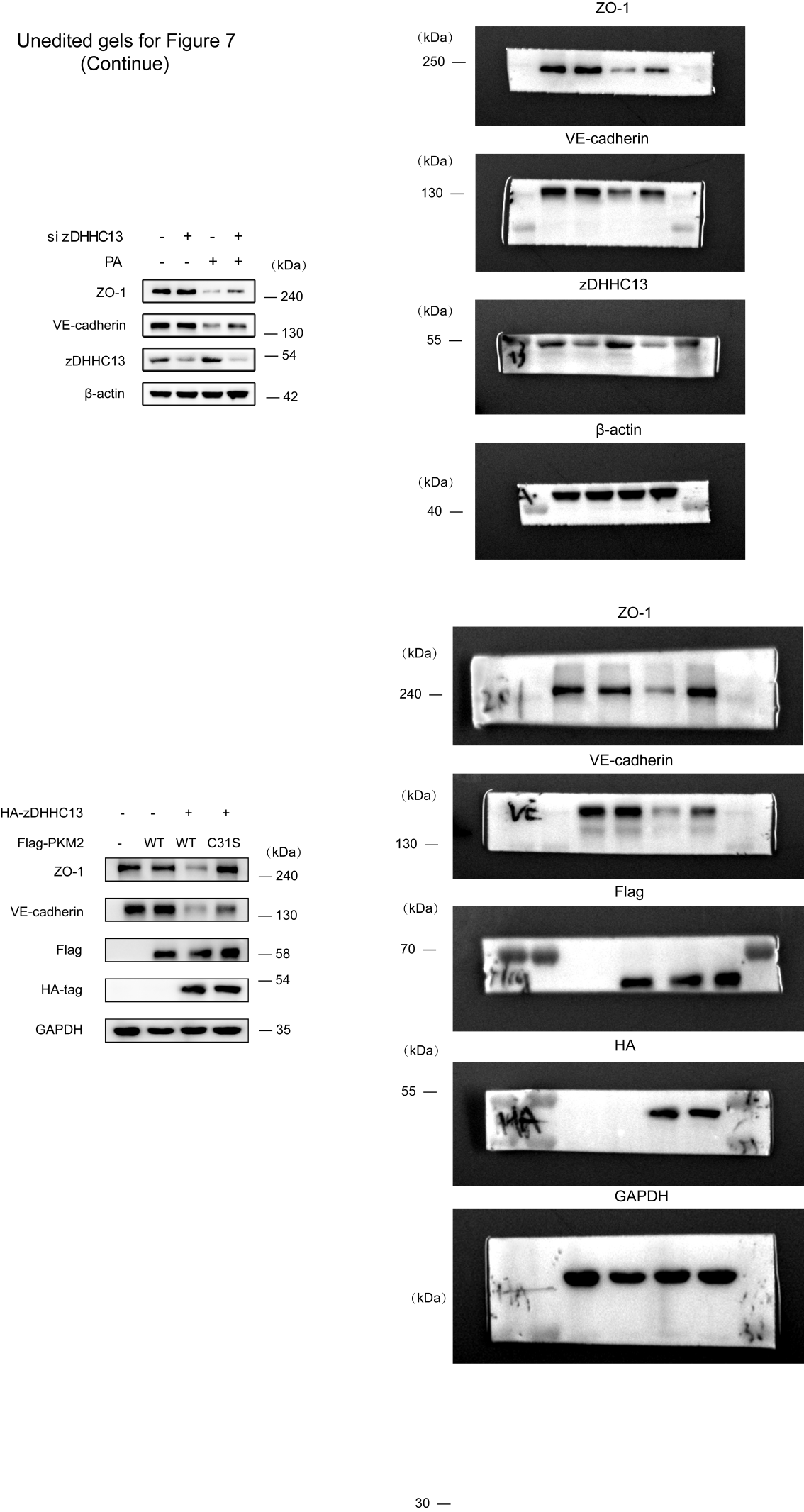

Supplement: Supplementary file 1 — Supporting Information [file ADVS-12-2412895-s001.docx]
